# JAK1/2 inhibition promotes aggressive cutaneous carcinogenesis through COT kinase–mediated alternative MAPK pathway activation

**DOI:** 10.1172/JCI199141

**Published:** 2026-11-02

**Authors:** Hélène Pasquer, Antoine Forget, Maxime Battistella, Raphael Marie, Léa Provent, Barouyr Baroudjian, Emmanuelle Latour, Cécile Culeux, Blandine Roux, Khansa Saadallah, Amandine Gouverneur, Maxime Demourgues, Fanélie Jouenne, Jérôme Lambert, Bérangère Lombard, Damarys Loew, Sophie Tartare-Deckert, Lionel Adès, Emmanuel Raffoux, Bruno Cassinat, Juliette Soret-Dulphy, Pernelle Noize, Samia Mourah, Camille Lobry, Jean-Jacques Kiladjian, Alexandre Puissant, Céleste Lebbé, Lina Benajiba

**Affiliations:** 1 INSERM UMR 1342, Institut de Recherche Saint-Louis, Paris, France.; 2 Université Paris Cité, INSERM Clinical Investigation Center (CIC) 1427, AP-HP, Hôpital Saint-Louis, Paris, France.; 3 Quantitative Biosciences Institute and; 4 Department of Cellular and Molecular Pharmacology, University of California, San Francisco, San Francisco, California, USA.; 5 Service de Pathologie, INSERM U976, Institut de Recherche Saint-Louis, and; 6 Dermato-oncology Unit and CIC, Cancer Institute AP-HP.Nord Paris Cité, INSERM U976, Université Paris Cité, AP-HP, Hôpital Saint-Louis, Paris, France.; 7 Université de Bordeaux, INSERM, BPH, U1219, AHeaD Team, CHU de Bordeaux, Pôle de Santé Publique, Service de Pharmacologie Médicale, Bordeaux, France.; 8 Service de Génomique des Tumeurs et Pharmacologie and; 9 Service de Biostatistiques, Université Paris Cité, AP-HP, Hôpital Saint-Louis, Paris, France.; 10 Université Paris Sciences et Lettres, Institut Curie, CurieCoreTech Spectrométrie de Masse Protéomique, Paris, France.; 11 Université Côte d’Azur, INSERM U1065 (C3M), Microenvironment, Signaling and Cancer, Nice, France.; 12 Département d’Hématologie et d’Immunologie and; 13 Laboratoire de Biologie Cellulaire, Université Paris Cité, AP-HP, Hôpital Saint-Louis, Paris, France.

**Keywords:** Dermatology, Hematology, Oncology, Clinical practice, Drug therapy, Skin cancer

## Abstract

Secondary malignancies are the most feared adverse effects of cancer therapies. In this study, we explored the cutaneous oncogenic effects of the JAK inhibitor ruxolitinib. First, we demonstrated an epidemiological association between ruxolitinib exposure and development of aggressive cutaneous squamous cell carcinoma (cSCC) by analyzing international pharmacovigilance data and conducting a large cohort study. We next demonstrated the causal link between ruxolitinib treatment and cSCC development using in vitro and in vivo models. Mechanistically, we revealed that ruxolitinib induces transformation of premalignant keratinocytes by activating the MAPK pathway. This effect is mostly driven by JAK2 inhibition, suggesting a pharmacological class effect. To unravel the mechanistic underpinnings of ruxolitinib-induced MAPK activation, we then applied a phosphoproteomic approach. Alternative MAPK signaling downstream of the kinase COT was pinpointed as the main activator of the pathway in this context. The central role of COT was functionally validated using chemical inhibition, genetic impairment, and inactivated COT mutant expression. Indeed, impairment of COT rescued the pro-oncogenic effect of ruxolitinib. Finally, we determined using a combination of epidemiological, in vitro, and in vivo studies that ruxolitinib-mediated oncogenic effects also result in an increased risk of melanoma, suggesting that the uncovered cellular signaling mechanism might be extrapolated to other cancers with a high dependency on the MAPK pathway.

## Abstract 2

JAK inhibitors could induce aggressive skin secondary cancers by activating a signaling proliferative pathway, the MAPK pathway.

## Introduction

Finding cancer cell–specific dependencies is the ultimate goal of cancer therapeutic research. However, exclusively targeting tumor cells remains challenging, and most current therapies also affect healthy tissues, leading to adverse events with various severity grades. Therapy-related malignancies, often developed years after exposure to cancer treatment, are the most feared adverse effects. While such events were initially described in the pediatric population characterized by long-term survival (1), their reported incidence has increased over the past decades in adults (2). This effect is thought to be driven by an increased number of long-term cancer survivors as a result of more efficient therapies. Indeed, curative treatment options are offered to an increasingly aged population, intrinsically harboring risk factors associated with higher incidence of malignancies. A subsequent challenge, therefore, is how to define therapy-related malignancies in populations with an already increased risk of cancer, driven by lifestyle habits and/or genetic predispositions (3). Beyond large-scale epidemiological studies, comprehensive laboratory-based mechanistic studies are needed to establish causality.

Secondary malignancies can emerge from different systemic cancer treatment types, resulting from diverse underlying mechanisms. Hematological malignancies, mainly myelodysplastic syndromes and therapy-related acute myeloid leukemia, can result from chemotherapy- and radiotherapy-mediated genomic instability or chromosomal translocation (4). Posttransplant lymphoproliferative disorders induced by EBV reactivation can emerge from the immune-suppressed state associated with hematopoietic stem cell transplantation (5). More recently, secondary T cell malignancies, thought to be driven by insertional mutagenesis, have been described after CAR T cell therapy (6, 7).

The current search for novel therapies is challenged by an increased demand for better treatments with improved benefit/risk ratios. Over the past decades, targeted therapies against cancer cell–specific oncogenic dependencies created the possibility for cancer treatments harboring lower toxicities (8). Although targeted therapies are usually nonmutagenic agents, as opposed to chemotherapy or radiation therapy, they have also been recently associated with an increased risk of therapy-related malignancies. Such secondary cancers could result from the selection of specific preexisting clones and the remodeling of their oncogenic potential. An example of such an oncogenic process comes from the recently described therapy-related acute myeloid leukemia secondary to PARP inhibitor treatment, which is thought to emerge from preexisting clones within the bone marrow harboring altered DNA damage repair pathways (9). Another example is the development of cutaneous squamous cell carcinoma (cSCC) after BRAF inhibitor exposure. Indeed, while BRAF inhibitors display anti-oncogenic effects on BRAF mutated melanoma cells, whose fitness relies on RAF/MEK/ERK signaling, they mediate RAF dimer transactivation, subsequently enhancing ERK signaling and resulting in pro-oncogenic effects in BRAF WT healthy keratinocytes (10, 11). In line with these observations, and given the increasing chronic exposure to many novel targeted therapies, long-term oncogenic effects of such molecules should be closely monitored in the coming years to improve cancer patient survival.

Clinical applications of JAK inhibitors have increased markedly over the past decade (12, 13), making it important to study long-term oncogenic effects that might emerge from this pharmacological class. Ruxolitinib was the first FDA-approved JAK1/2 inhibitor, initially developed to impair JAK/STAT pathway hyperactivation in myeloproliferative neoplasms (MPNs) (14, 15). Due to its immunosuppressive properties, ruxolitinib is now also approved for the treatment of post-hematopoietic stem cell transplantation graft-versus-host disease (GvHD) and a variety of inflammatory/autoimmune disorders (16, 17). During the first decade of its commercial use, ruxolitinib exposure has been associated with a 15-fold increased risk of aggressive B cell lymphoma (18) and an increased incidence of non-melanoma skin cancers (19). Several clinical observations confirmed the latter finding, with reports of aggressive cSCC cases developed under ruxolitinib treatment in patients treated for MPN (20–23) or GvHD (24). These observations warrant further epidemiologic and mechanistic exploration to better delineate the long-term oncogenic effects potentially mediated by ruxolitinib.

In the present study, we explore ruxolitinib effects on cutaneous oncogenesis. We first validate the epidemiological association between ruxolitinib exposure and increased risk of cSCC, combining an international pharmacovigilance study with a large cohort study. We next demonstrate the causal link between ruxolitinib treatment and cSCC development, using in vitro and in vivo models. Using genetic and chemical tools, we show that this oncogenic effect is driven by JAK1 and/or JAK2 impairment, suggesting a JAK inhibitor pharmacological class effect. Mechanistically, we reveal that JAK/STAT pathway inhibition results in COT kinase–mediated alternative MAPK pathway activation, underlying the observed oncogenic effects. Beyond cSCC, using in vitro and in vivo models combined with an epidemiological study, we show that ruxolitinib exposure leads to similar oncogenic effects in melanoma, another MAPK-driven skin cancer.

## Results

### Ruxolitinib exposure is associated with the development of aggressive cSCC.

To evaluate the impact of ruxolitinib on cSCC development, we first conducted an international pharmacovigilance study using VigiBase, WHO’s database centralizing adverse drug reaction cases spontaneously reported to national pharmacovigilance systems. Among 4,409,093 cases involving antineoplastic and immunomodulating agents, ruxolitinib was suspected in 34,234, including 76 cSCC cases (median age, 73 years; women, 13.2%) (Figure 1A). Most cases (99%) were serious; 8% led to patient death, and 21% led to hospitalization or hospitalization prolongation (Figure 1B and Supplemental Table 1; supplemental material available online with this article; https://doi.org/10.1172/JCI199141DS1). When considering all antineoplastic and immunomodulating agents as the reference group, signals of disproportionate reporting for cSCC were found for ruxolitinib through both disproportionality measures: Proportional Reporting Ratio (PRR) 4.88 _95%CI_[3.88;6.14] and Information Component (IC) 2.21 [IC_025_ 1.83]. Analyses using other drugs with the same indication as a comparator confirmed the specific association between ruxolitinib and cSCC (Figure 1C and Supplemental Table 2).

To confirm this association, we interrogated a local cohort of 1,004 patients with MPN, including 136 patients treated with JAK inhibitors (Figure 1D and Supplemental Table 3). Fifteen patients treated with JAK inhibitors developed cSCC (11.0%), whereas only 14 patients not treated with a JAK inhibitor (1.6%) developed cSCC. Median time between JAK inhibitor treatment initiation and cSCC development was 31 months, _range_[1;80]. Twenty-year cSCC cumulative incidence was 13% for MPN patients treated with JAK inhibitor compared with 3% in the absence of JAK inhibitor (*P* < 0.001; Figure 1E). In multivariable analysis, JAK inhibitor treatment (HR = 6.52, _95%CI_[2.75;15.46], *P* < 0.001) was associated with cSCC development, independently of age (HR = 1.04, _95%CI_[1.02;1.06], *P* = 0.001) and male sex (HR = 3.52, _95%CI_[1.39;8.93], *P* = 0.008), which are known cSCC risk factors (Figure 1F and Supplemental Table 4). Among the 15 patients with cSCC developed under JAK inhibitor treatment, 6 (40%) presented aggressive cSCC (locally advanced, with lymph node infiltration and/or pulmonary metastasis), while all patients with JAK inhibitor–naive cSCC had localized disease (Figure 1G). Clinical and histological aggressiveness criteria are reported in Figure 1H and Supplemental Table 5.

### Ruxolitinib treatment promotes aggressive cSCC in in vitro and in vivo models.

After demonstrating the epidemiological association between aggressive cSCC development and ruxolitinib, we used in vitro and in vivo models to evaluate the causal link underlying this clinical observation. In vitro, the A431 cSCC human cell line was resistant to high doses of ruxolitinib (Figure 2A). More interestingly, we observed an overgrowth of A431 cells under increasing doses of ruxolitinib, up to 12.5 μM (Figure 2B). We next used a chemically induced cSCC murine model to validate this effect in vivo and investigate whether ruxolitinib exposure results in an increased rate of premalignant-to-malignant transformation. This model resulted in the development of premalignant lesions (papillomas), of which a subset spontaneously evolved into overt cSCC (Figure 2C). At 22 weeks after first chemical agent application, mice were randomized into 2 groups (*n* = 9 per group) and treated with vehicle or ruxolitinib. Six weeks after treatment initiation, 11 and 5 cSCC tumors developed in the ruxolitinib and vehicle groups, respectively. cSCC incidence was higher after ruxolitinib exposure (9/9 versus 3/9) (Figure 2D). Median number of premalignant lesions was not different before (23 _range_[10;40] versus 22 _range_[13;41]) and after treatment (22 _range_[12;47] and 24 _range_[12;37]), in vehicle and ruxolitinib groups, respectively (Figure 2E). This absence of difference between both groups suggests that ruxolitinib effects on cSCC development result from an increased transformation rate of precancerous lesions, rather than de novo oncogenic lesions development. In addition to increasing the incidence of cSCC, ruxolitinib also promoted more aggressive tumors, therefore mimicking our patient cohort observations. Median tumor volume per mouse was bigger in the ruxolitinib group (vol = 337.3 mm^3^_range_[147;720] versus vol = 114.6 mm^3^_range_[49;800]) (Figure 2F). Moreover, 2 ruxolitinib-exposed mice developed lymph node and lung metastasis, while no metastasis was observed in the control group (Figure 2G). Ruxolitinib-promoted cSCC also presented histological aggressiveness criteria (poor differentiation, necrosis, vascular embolism, and histological metastasis) (Figure 2, G–I, and Supplemental Table 6).

Altogether, our data highlight the key causal role of ruxolitinib exposure in cutaneous oncogenesis, resulting in the promotion of aggressive secondary cSCC from precancerous lesions.

### MAPK pathway activation drives ruxolitinib-mediated cutaneous carcinogenesis.

We next explored the underlying mechanisms involved in ruxolitinib-mediated aggressive oncogenesis. GSEA of transcriptomic data obtained from control murine carcinomas (*n* = 3) compared with murine carcinomas developed under ruxolitinib treatment (*n* = 4) decreased JAK/STAT signaling in ruxolitinib-mediated tumors (Figure 3A). This analysis also showed that carcinoma oncogenic pathways, skin/keratinocyte proliferation pathways, and pathways involved in poor tumor differentiation were enriched in ruxolitinib tumors, in line with the increased aggressiveness of ruxolitinib-mediated cSCC (Figure 3A). Interestingly, among oncogenic signaling pathways known to be involved in cSCC proliferation, we observed an upregulation of the MAPK pathway in ruxolitinib tumors (Figure 3, A–C). Accordingly, an increased ERK phosphorylation level was detected by immunofluorescence in mice tumors developed under ruxolitinib treatment (Figure 3D). In the ruxolitinib-treated A431 cSCC cell line, ERK phosphorylation level, evaluated by Western blot, was also increased, while STAT3 and STAT5 phosphorylation was decreased (Figure 3E).

To demonstrate the key functional role of MAPK pathway activation in ruxolitinib-mediated cutaneous oncogenesis, we next evaluated the effect of MEK inhibition. In vitro, addition of the MEK inhibitor (MEKi) CI-1040 at its IC_50_ (Figure 3F) reduced the ruxolitinib-induced increase in ERK phosphorylation (Figure 3G) and cell growth (Figure 3H). In vivo, the addition of CI-1040 to ruxolitinib reduced both cSCC incidence and mean tumor volume (Figure 3, I and J).

To further validate the clinical relevance of MAPK pathway hyperactivation in ruxolitinib-mediated cSCC oncogenesis in patients, we compared ERK phosphorylation levels in 5 aggressive cSCC tumors developed under ruxolitinib to 5 spontaneously developed cSCC, matched according to TNM (tumor, node, metastasis) stage and patient age. Immunochemistry quantification confirmed the increase of ERK phosphorylation level in tumors developed under ruxolitinib (Figure 3K).

### Ruxolitinib-mediated cutaneous carcinogenesis results mostly from JAK2 inhibition and represents a pharmacological class effect.

Determining whether ruxolitinib-mediated aggressive cSCC is specific to first-generation JAK inhibitors and whether it is related to JAK1 and/or JAK2 inhibition are key clinical objectives. Indeed, many JAK inhibitors are now available for the treatment of MPN and nonmalignant inflammatory and autoimmune disorders. Moreover, whereas ruxolitinib inhibits both JAK1 and JAK2, more selective JAK2 inhibitors, with minimal to no activity against JAK1, have recently become available for the treatment of myelofibrosis. To evaluate the pro-oncogenic effect of JAK inhibitors, we reinterrogated WHO’s pharmacovigilance database to obtain the number of cSCC cases notified over time between 2014 and 2025 under 7 different JAK inhibitors (ruxolitinib, tofacitinib, baricitinib, fedratinib, pacritinib, momelotinib, and itacitinib). The number of reported cases under ruxolitinib and tofacitinib, the oldest JAK inhibitors commercialized since 2012, increased over time (7–193 for ruxolitinib and 3–73 for tofacitinib between 2015 and 2025). However, the magnitude of increase was lower with tofacitinib. For more recently approved JAK inhibitors, such as baricitinib, fedratinib, pacritinib, and momelotinib, a lower number of cutaneous squamous cell carcinoma cases have been reported (17, 1, 1, and 0, respectively) (Supplemental Figure 1 and Supplemental Table 7).

To further assess the potential pro-oncogenic effects of different JAK inhibitors, we subsequently evaluated the effect of JAK1 or JAK2 genetic impairment, as well as the effect of 7 JAK inhibitors (ruxolitinib, fedratinib, itacitinib, momelotinib, pacritinib, tofacitinib, and baricitinib) harboring diverse JAK1/2 selectivity, on cSCC proliferation and signaling in vitro. First, to explore if ruxolitinib-mediated cutaneous carcinogenesis results from JAK1 or JAK2 inhibition, we used genetic inhibition of JAK1 or JAK2. JAK1 genetic impairment using 4 JAK1-directed shRNAs downregulated JAK1 expression without affecting JAK2 expression (Figure 4A) and decreased downstream P-STAT3 signaling (Figure 4B). Genetic inhibition of JAK1 led to an increase of MAPK pathway signaling (Figure 4B) and a mild excess of A341 cell proliferation compared with control shRNA (~1.25- to 1.5-fold) (Figure 4C). JAK2 genetic inhibition downregulated JAK2 expression without affecting JAK1 expression (Figure 4D) and decreased downstream P-STAT5 signaling (Figure 4E). More markedly than JAK1 genetic inhibition, JAK2 genetic impairment using 4 JAK2-directed shRNAs increased P-ERK expression (Figure 4E) and led to an increased cell proliferation of A431 compared with control shRNA (~2- to 5-fold) (Figure 4F).

For more clinical relevance, we confirmed these results using chemical inhibition. In vitro, JAK1/2 inhibitors (ruxolitinib, momelotinib, baricitinib, and tofacitinib), JAK2-specific inhibitors (fedratinib and pacritinib), and a JAK1-specific inhibitor (itacitinib) increased proliferation of A431 cells (Figure 5A). All JAK inhibitors increased P-ERK expression (Figure 5B) compared with DMSO. The JAK1 inhibitor itacitinib showed the mildest cell proliferation increase, in line with a lower increase in ERK phosphorylation (Figure 5, A and B). In vivo, using our chemically induced mouse model, we demonstrated that the number of precancerous lesions in mice treated with fedratinib, a specific JAK2 inhibitor, was not different from control mice (Figure 5C). However, both the number and volume of cSCC were increased in mice treated with fedratinib compared with control (Figure 5, D and E). Finally, in our MPN cohort, among all cSCCs associated with JAK inhibitors, 86%, 7%, and 7% were related to ruxolitinib, pacritinib, and momelotinib, respectively (Figure 5F). Altogether, these results suggest that the cutaneous pro-oncogenic effect of ruxolitinib is primarily driven by JAK2 inhibition and may be observed across all JAK2 inhibitors, including more recently approved agents.

### Ruxolitinib-induced MAPK pathway activation is mediated by the COT kinase.

To better understand the mechanism underlying ruxolitinib-induced MAPK activation, we first evaluated the molecular landscape of ruxolitinib-mediated cSCC in patients since RAS pathway mutations are key drivers of cSCC oncogenesis. Next-generation sequencing (NGS) of 6 aggressive cSCCs developed under ruxolitinib treatment for MPN management revealed a high incidence of *TP53* mutations (5/6), in line with the genomic landscape of non–ruxolitinib-mediated cSCC (25). Mutations in *HRAS*, known to be the most frequently mutated *RAS* isoform in cSCC, were not observed (Supplemental Figure 2, A and B). Within the rest of the RAS pathway genes, only 1 *NRAS* variant, *c.419C>T* p.P140L, of uncertain significance was detected in 1 tumor. Of note, *JAK2* V617F mutation, the main driver of MPN, was detected with a low variant allele frequency (1%, 8.7%, and 2.2%) in cSCC obtained from 3 patients diagnosed with polycythemia vera. *JAK2* V617F allelic burden was very high (90%, 95%, and 87%) in peripheral blood for all of them, suggesting minimal blood contamination rather than carcinoma cells harboring this mutation (Supplemental Figure 2C). Altogether, these findings argue for a nongenetic mechanism for MAPK activation in ruxolitinib-mediated cSCC.

Thus, to better understand the mechanism underlying MAPK pathway activation under ruxolitinib treatment, we first performed an unbiased proteomic analysis in mice tumors associated with ruxolitinib (*n* = 5) compared with control tumors (*n* = 4). Among the 5,743 proteins quantified, 1,100 proteins were differentially expressed between ruxolitinib and control tumors (Supplemental Table 8). GSEA of differential protein levels between ruxolitinib- and vehicle-treated tumors showed significant enrichment of cell cycle– and RTK signaling–related Gene Ontology terms in ruxolitinib-treated tumors, in line with keratinocyte proliferation induced by MAPK pathway hyperactivation in the context of ruxolitinib treatment (Supplemental Figure 2D). Cell cycle analysis of the ruxolitinib-treated A431 cell line confirmed the increased cell cycling of cSCC cells in the context of ruxolitinib treatment, with an increased proportion of cells in S and G_2_/M compared with DMSO-treated cells (Supplemental Figure 2E). Moreover, the addition of MEKi treatment rescued the effect of ruxolitinib on the cell cycle (Supplemental Figure 2E). Additionally, a phosphoproteomic analysis in mice tumors associated with ruxolitinib (*n* = 5) compared with control (*n* = 4) revealed that among the 9,654 phosphosites identified, 1,930 (corresponding to 801 proteins) differed between ruxolitinib and control tumors (Supplemental Table 9).

We next performed kinase enrichment analysis (KEA) (26) using differentially abundant phosphosites between ruxolitinib and control tumors. This analysis showed an enrichment of cell cycle–related kinases, such as CDK1/2, as well as multiple kinases downstream of MAP3Ks, such as ERK1/2, JNK1/2/3, and P38A/B/D (Figure 6A). This observation confirms the likely activation of the MAPK pathway in tumors treated with ruxolitinib. However, RAS and RAF activating phosphosites, known to be involved in the classical path toward MAPK pathway activation, were not enriched in the context of ruxolitinib exposure, thereby indicating a noncanonical mechanism of MAPK activation by ruxolitinib (Supplemental Table 10). Alternative kinase prediction tools (27) suggested an activation of the MAP3K COT (also named TPL2 or MAP3K8) (Figure 6B). Interestingly, COT has been shown to be an upstream activator of ERK, JNK, and P38 kinases, which is consistent with the broad activation of these kinases observed by KEA (Figure 6C). COT is an oncoprotein kinase shown to play a key role in melanoma resistance to BRAF inhibitors (28). Based on the previously described role of COT (28) and our phosphoproteomic results, we hypothesized that COT is key for alternative MAPK pathway activation, subsequently resulting in ruxolitinib-mediated aggressive cSCC. To validate this hypothesis, we first confirmed that the COT Thr290 phosphosite, known to be an activating phosphosite in the activation loop of the kinase domain of COT (29), was increased under ruxolitinib treatment of the A431 cSCC cell line, while RAF phosphorylation was decreased (Figure 6D). To explore the functional impact of COT kinase activation on ruxolitinib-mediated cSCC proliferation, we used 2 small-molecule COT inhibitors (TPL2 kinase inhibitor 1 and COT inhibitor 2) at their IC_50_ (Figure 7A). Addition of either COT inhibitor to ruxolitinib reduced the ruxolitinib-induced hyperproliferation phenotype (Figure 7B). Similarly, COT genetic impairment using 3 *COT*-directed shRNAs (Figure 7C) also dampened the ruxolitinib-induced pro-proliferative effects on cSCC cells (Figure 7D). Finally, to evaluate whether COT phosphorylation in Thr290 is critical for the ruxolitinib effect, we transduced the A431 cell line with (a) an shRNA against COT to block endogenous COT and (b) an exogenous codon-optimized WT or threonine-to-alanine (T290A) mutant form of COT. ERK phosphorylation and A431 cell proliferation increased upon ruxolitinib exposure in COT-WT–expressing cells (Figure 7, E and F). Interestingly, expression of the T290A-COT mutant impaired ruxolitinib-mediated ERK phosphorylation increase and blocked its effect on cell proliferation, suggesting that COT T290 phosphorylation is critical for ruxolitinib-mediated MAPK activation and cSCC cell proliferation. Overall, our results pinpoint a COT-mediated alternative MAPK pathway activation mechanism as being responsible for ruxolitinib-mediated aggressive cSCC development.

### Ruxolitinib-induced COT kinase activation involves a posttranslational regulation mechanism.

We next set to further explore the link between JAK inhibition and COT activation. To get more mechanistic insight, we explored 2 hypotheses. Indeed, JAKs are known to impact cellular proliferation through at least 2 distinct mechanisms: transcriptional regulation through the STAT downstream factors or more directly through modulating protein phosphorylation. We thus first evaluated the effect of JAK inhibition on COT transcription in vitro in the A431 cSCC cell line. COT transcript levels were similar between ruxolitinib and control cells, as shown by RT-qPCR at 3 different time points (Figure 8A). We next hypothesized that phosphorylation changes under JAK inhibitor treatment might be involved in COT activation. In line with this hypothesis, we observed that phosphorylation of COT at T290 is an early event occurring only 15 min after ruxolitinib treatment, suggesting that ruxolitinib-mediated COT activation involves a posttranslational mechanism (Figure 8B). COT posttranslational regulation is still largely unknown. We hypothesized that, under basal conditions, JAKs phosphorylate and activate a phosphatase, which in turn dephosphorylates COT at T290, thereby reducing its activity. To test this hypothesis, we examined COT and ERK phosphorylation as well as cSCC proliferation in the presence of a phosphatase inhibitor known to target threonine and serine phosphatases. At all tested doses, threonine/serine phosphatase inhibition recapitulated the effects observed with ruxolitinib, resulting in increased COT phosphorylation at T290, enhanced ERK phosphorylation, and increased cell proliferation (Figure 8, C and D). Taken together, these results suggest that ruxolitinib might repress a COT-inactivating phosphatase, thereby promoting activation of the MAPK pathway and enhancing cell proliferation (Figure 8E).

### Ruxolitinib-mediated oncogenic effects result in an increased risk of melanoma.

Melanoma is another skin cancer type exhibiting a high dependency on the MAPK pathway (30). Given ruxolitinib pro-oncogenic effects mediated by MAPK pathway activation and resulting in an increased risk of aggressive cSCC, we further explored ruxolitinib effects on melanoma. In vitro, using 2 different melanoma cell lines (B16-OVA and YUMMER1.7), we showed that ruxolitinib-induced cell proliferation compared with DMSO conditions (Figure 9A). We then confirmed these results in vivo. Indeed, in 2 different melanoma mouse models (B16-OVA and YUMMER1.7), tumor volume change from baseline was higher in the ruxolitinib-exposed mice group compared with the control group after 10 days of treatment (Figure 9B). Finally, we interrogated our MPN cohort to evaluate melanoma incidence. Importantly, melanoma incidence was higher for patients who received JAK inhibitor treatment (2.9% versus 0.35%) (Figure 9C). Altogether, these results suggest a potential role of ruxolitinib exposure in melanoma oncogenesis, and further epidemiological and mechanistic studies should explore this finding and determine clinical guidelines for patients exposed to JAK inhibitors.

## Discussion

Human tissues are a complex mosaic of different genomes (31). Therefore, targeting a specific oncogenic pathway in one tissue could result in increased fitness and secondary malignancy in another one composed of cells harboring divergent cell fitness dependencies. In this context, we explored ruxolitinib oncogenic effects on cutaneous tissue. While showing beneficial therapeutic effects in MPN, JAK/STAT pathway inhibition has been recently suggested to favor cSCC development (32). We confirmed this risk association by combining 2 independent exhaustive epidemiological studies. Our extensively annotated MPN cohort pinpointed the higher aggressiveness of ruxolitinib-associated cSCC. Indeed, 40% of cSCCs developed under JAK inhibitor treatment were locally advanced or metastatic, while only 3%–5% of reported cSCCs in the literature harbor such aggressiveness criteria (33). These findings are in line with the study by Rampotas et al. (34), in which 90 cases of non-melanoma skin cancers with aggressive features and high mortality were reported in ruxolitinib-treated patients.

Epidemiological association studies are not suited to confirm causation, as confounding factors could be responsible for the association between ruxolitinib exposure and an increased risk of cSCC. To overcome such bias and establish a causal link between treatment exposure and secondary cancer development, in vivo models remain the gold standard (35). Taking advantage of a chemically induced cSCC murine model, we demonstrated for the first time, to our knowledge, the causal link between ruxolitinib exposure and cSCC development. Moreover, our results pinpointed the underlying mechanism as an acceleration of the premalignant-to-malignant lesion transition, in contrast to the oncogenic initiating effects of genotoxic treatments, such as hydroxyurea (36). This mechanism is reminiscent of aggressive B cell lymphoma development under ruxolitinib treatment, which is thought to emerge from preexisting clonal immunoglobulin gene rearrangements within a patient’s bone marrow (18).

Mechanistically, we demonstrated that ruxolitinib-associated cutaneous carcinogenesis is mediated by MAPK pathway activation. MAPK activation is classically driven by RAS and RAF signaling (37). Interestingly, ruxolitinib-associated MAPK activation was not driven by *RAS* mutations, in line with our previous results suggesting that ruxolitinib accelerates the transformation of premalignant lesions rather than generating new mutations. Moreover, the MPN driver clone (*JAK2* V617F) was not found in ruxolitinib-mediated skin tumors. In line with these findings, B cell lymphomas that developed under ruxolitinib treatment did not harbor MPN-associated mutations (18). Additionally, Lee et al. recently reported a case of aggressive cSCC that developed in a patient treated with ruxolitinib for GvHD, suggesting that such secondary carcinogenesis occurs independently from the MPN context (24). To maintain the key MAPK pathway activation necessary for their proliferative advantage in the absence of RAS mutations, ruxolitinib-treated keratinocytes actioned a COT-mediated alternative path. The serine/threonine kinase COT is known to directly phosphorylate MEK (28). Indeed, COT expression drives resistance to BRAF inhibitors in melanoma (28), and COT/MAP3K8 rearrangements recurrently occur in spitzoid melanoma (38). Altogether, our findings highlight the divergent fitness effects of JAK/STAT pathway targeting on hematopoietic versus cutaneous tissues, in line with their different oncogenic dependencies. Indeed, the MAPK pathway plays a key role in normal and malignant keratinocytes’ proliferation, while hematopoietic cells mainly rely on the JAK/STAT pathway for their proliferative signaling (39–41). We further speculate that our results could be extended to other tissues with a high dependency on the MAPK pathway.

In terms of clinical implications for patients treated with JAK inhibitors, our findings argue for dermatological screening for all patients treated with JAK2 inhibitors. Drug exposure duration, number of treated patients, and skin cancer risk profile of treated patients may account for the observed differential skin cancer effects of diverse JAK inhibitors in the real-world setting. Indeed, our study shows that cutaneous cancers develop after a median of 31 months after ruxolitinib initiation. We can thus not exclude that a higher incidence of secondary skin cancers might be observed with more recently approved JAK inhibitors after a longer post-commercialization follow-up and a larger number of treated patients. Moreover, ruxolitinib is primarily used to treat patients with MPNs, which harbor an increased risk of secondary cancers (42). Indeed, MPN is diagnosed at a median age of approximately 65 years old, and patients with MPN harbor an immune-dysregulated profile resulting from the hematological disease and long-term exposure to anticancer therapies (43, 44). This patient profile is thus more prone to develop preneoplastic skin lesions and subsequently transform them into overt cutaneous squamous cell carcinomas. While baricitinib and tofacitinib are widely used, patients for whom these inhibitors are indicated — mainly in rheumatology to treat inflammatory and autoimmune disorders — harbor a lower risk of preneoplastic skin lesions. In line with this, since Ytterberg et al. (45) reported an increased risk of secondary malignancies under tofacitinib treatment in patients treated for rheumatoid arthritis, the indication of JAK inhibitors for arthritis treatment was limited in patients older than 65 years. The lower risk of precancerous lesions and overt cancers of this population might explain why fewer secondary neoplasms have been observed with more recently approved JAK inhibitors for nonmalignant indications, such as baricitinib. Patients also harboring other cSCC risk factors (e.g., age, male sex, actinic keratosis, or cSCC history) should be closely monitored, in line with our data demonstrating the role of ruxolitinib in accelerating the premalignant-to-malignant transition. JAK inhibitors can be used at different doses according to their indication, tolerance, and efficacy. Thus, assessing if there is a JAK inhibitor dose effect on carcinogenesis is important. However, accurately assessing the doses received by the patients included in our epidemiological study is challenging in this retrospective setting. Indeed, current clinical practice for MPN patient management requires repeated ruxolitinib dose adjustments to obtain efficacy while managing toxicity (mainly cytopenias). As aggressive cutaneous carcinomas induced by ruxolitinib were more frequent in elderly patients (median age: 71.5 years), we can speculate that low/intermediate doses of ruxolitinib (10 mg twice daily [bid] or even 5 mg bid) are sufficient to promote carcinogenesis. Indeed, in elderly patients, ruxolitinib doses are often reduced due to hematological toxicities. Our in vitro data are in line with this hypothesis, as we did not observe a clear/consistent dose effect on cSCC proliferation increase. Indeed, across the large panel of ruxolitinib doses evaluated — ranging from 0.097 to 12.5 μM — we observed an excess of cSCC proliferation at all tested doses compared with DMSO, suggesting a threshold rather than a dose effect on carcinogenesis, as the lowest dose tested is sufficient to induce the observed effect. Our results with the 6 other JAK inhibitors evaluated in vitro across a large panel of doses — ranging from 0.097 to 12.5 μM — are also in line with a threshold effect, as cellular proliferation increased at the lowest evaluated dose and did not further increase with dose increase. Interestingly, for some of the tested inhibitors (fedratinib, pacritinib, and momelotinib), the highest doses decreased cellular proliferation. We hypothesize that this might be related to off-target inhibition of other kinases at the highest tested doses. Further validation with prospective patients’ data would strengthen this hypothesis.

Finally, for patients with a risk of cSCC development and treated with JAK inhibitor or for patients who develop JAK inhibitor–mediated aggressive cSCC, addition of MEKi seems to be an efficient preventive or curative therapeutic strategy, enabling maintenance of JAK inhibitor treatment for MPN disease management. Discontinuation of JAK inhibitor treatment is indeed highly challenging in the absence of therapeutic alternatives (46). The clinical benefits of using MEKi to counteract JAK inhibitor–mediated, COT-driven MAPK pathway activation should be evaluated in a clinical trial.

Importantly, we show using a combination of epidemiological, in vitro, and in vivo studies that ruxolitinib-mediated oncogenic effects also result in an increased risk of melanoma, another skin cancer with a high dependency on the MAPK pathway. However, because the incidence of melanoma was low in our MPN cohort; further validation in larger cohorts is needed to confirm the association between melanoma development and ruxolitinib treatment. Nonetheless, our findings suggest that ruxolitinib exposure could increase the risk of developing other secondary cancers with a high dependency on the MAPK pathway. Indeed, Ytterberg et al. showed that exposure to tofacitinib, used to treat inflammatory diseases, is associated with an increased risk of cancers other than non-melanoma skin cancers, including lung adenocarcinomas, which are highly dependent on MAPK signaling (45). In our study, the high incidence of premalignant skin lesions (actinic keratosis) within the elderly MPN patient population led to the discovery of the role of ruxolitinib exposure in cSCC development. A lower frequency of premalignant lesions, combined with a potentially more efficient antitumor immune response in other organs, could preclude the identification of other epidemiological associations. Large, long-term, real-life, epidemiological studies are needed to unravel the spectrum of secondary cancers associated with JAK inhibitor exposure, particularly in the context of extended clinical use of JAK inhibitors for diverse nonmalignant inflammatory disorders.

Finally, ruxolitinib has a known immunosuppressive effect, mediated by inhibition of JAK1/2-dependent cytokine signaling. This effect is therapeutically exploited in the treatment of GvHD (47). While this immune-suppressive effect could play a role in skin cancer promotion and further explain the aggressiveness of ruxolitinib-associated cSCC, the immune effects of ruxolitinib might be different in the highly inflammatory and immune-suppressive tumor microenvironment context. Recently, Mathew et al. (48) and Zak et al. (49) demonstrated that JAK inhibitors enhance response to immune checkpoint inhibitors in lung adenocarcinoma and Hodgkin lymphoma, respectively. Indeed, JAK inhibition alleviated the immune-suppressive effects of these tumors. Whether ruxolitinib could reduce the immune-suppressive effects of cSCC in the context of ruxolitinib-mediated aggressive cSCC remains to be explored.

In conclusion, ruxolitinib induces the transformation of premalignant lesions into aggressive cSCC via MAPK pathway activation driven by COT kinase activation. This effect is mainly observed with JAK2 inhibitors and warrants careful dermatological monitoring for patients exposed to this pharmacological class. Beyond cSCC, our data suggest that JAK inhibitors can also result in an increased risk of other cancers highly dependent on the MAPK pathway, such as melanoma.

## Methods

### Sex as biological variable

For human studies, our study examined male and female individuals, and secondary skin cancers were observed within both sexes.

For animal models, our study examined female mice because female animals harboring skin tumors exhibited less aggressiveness. It is unknown whether the findings are relevant for male mice.

### International pharmacovigilance study

All cSCC cases (MedDRA preferred terms: cSCC metastatic, cSCC recurrent, and squamous cell carcinoma of skin) suspecting ruxolitinib were identified using VigiBase, WHO’s database centralizing adverse drug reaction cases spontaneously reported to national pharmacovigilance systems. Disproportionate reporting of cutaneous squamous cell carcinoma suspecting ruxolitinib was studied using 2 measures (PRR and IC), considering (a) all other antineoplastic and immunomodulating agents, (b) drugs with the same indication (e.g., hydroxycarbamide, interferon α-2a and -2b, peginterferon α-2a and -2b, and ropeg-interferon α-2b), and (c) hydroxycarbamide as the reference (50). PRR with a 95% CI lower limit exceeding 1 or IC with a 95% credibility interval lower limit (IC_025_) exceeding 0 indicated signals of disproportionate reporting. A second data interrogation was performed to evaluate the evolution of the number of cases notified over time (between 2014 and 2025) under different JAK inhibitors (ruxolitinib, tofacitinib, baricitinib, fedratinib, pacritinib, momelotinib, and itacitinib). Analyses were performed using SAS software, version 9.4 (SAS Institute).

### MPN patient cohort

#### Patient population.

A total of 1,004 consecutive patients were diagnosed with MPN according to WHO criteria and were followed in our MPN reference center at Saint-Louis Hospital between 2011 and 2020. Clinical, biological, and molecular characteristics at MPN diagnosis, cSCC diagnosis, and during follow-up were collected from medical charts and electronic medical records by specialized dermatologists and hematologists with expertise in cSCC and MPN management, respectively.

#### MPN and cSCC patient sample molecular data acquisition.

A custom-designed NGS capture panel including 40 genes involved in squamous carcinoma was used for the comprehensive genotyping of ruxolitinib-associated cSCC (Supplemental Table 11) (25, 51). DNA was extracted using the Maxwell RSC FFPE Plus DNA kit for FFPE tissue from five 10 μm sections according to the manufacturer’s protocol. After library production using SureSelect XT HS technology (Agilent), qualification of libraries was performed with BioAnalyzer/TapeStation (Agilent). Sequencing was conducted on a NextSeq 2000 platform (Illumina) with a custom bioinformatics pipeline detecting single-nucleotide and copy number variations. Confirmed or probable pathogenic genetic alterations were identified using at least 3 out of 5 pathogenicity predictive algorithms (CADD, SIFT, PolyPhen2, AlignGVGD, and Mutation Taster) and had a frequency in general population databases < 1%.

For MPN samples, we used a capture-based custom NGS panel (Sophia Genetics) targeting 36 myeloid genes, as previously described (52). Significant variants were retained with a sensitivity of 1%. Molecular data were illustrated using cBioPortal vv3.0.0 (https://www.cbioportal.org/).

*JAK2* variant allele frequency was quantified in blood and cSCC-extracted DNA using an allele-specific aPCR kit (*JAK2* MutaQuant Ipsogen, Qiagen). The amplification was performed using a QuantStudio 7 Flex instrument (Thermo Fisher Scientific).

#### Immunochemistry.

Five samples of aggressive cSCC developed under ruxolitinib treatment were matched by TNM stage and patient age with 5 samples of spontaneously developed cSCC diagnosed at Saint-Louis Hospital. FFPE tissue sections for each sample were immunostained using phospho-ERK1/2 antibody (Thr202/Tyr204; D13.14.4E clone, Ozyme) and analyzed on a BenchMark Ultra automated immunostainer (Roche). For quantification, pictures of 4 representative fields were taken using a Nanozoomer 2.0 (Hamamatsu) by a blinded operator. Positive areas of P-ERK of each picture were quantified using QuPath v0.5.1 (53), and mean positive area was calculated for each tumor.

### Cell culture

#### Cell lines and primary cells.

The A431 cSCC human cell line was purchased from the American Type Culture Collection (CRL-1555) and maintained in RPMI 1640 (Sigma-Aldrich) supplemented with 1% penicillin-streptomycin (Sigma-Aldrich) and 10% FBS (Sigma-Aldrich) at 37°C with 5% CO_2_.

B16-OVA murine melanoma cancer cells were provided in-house and maintained in DMEM high glucose (Sigma-Aldrich) supplemented with 1% penicillin-streptomycin (Sigma-Aldrich), 10% FBS (Sigma-Aldrich), 1% Glutamax (Thermo Fisher Scientific), and 1 mg/mL of Geneticin (Thermo Fisher Scientific) at 37°C with 5% CO_2_.

YUMMER 1.7 murine melanoma cancer cells were provided in-house and maintained in DMEM F12 (Sigma-Aldrich) supplemented with 1% penicillin-streptomycin (Sigma-Aldrich), 10% FBS (Sigma-Aldrich), and 1% Glutamax (Thermo Fisher Scientific) at 37°C with 5% CO_2_.

### Cell growth analysis

#### Chemicals.

Ruxolitinib (HY-50856), itacitinib (HY-16997), fedratinib (HY-10409), momelotinib (HY-10961), pacritinib (HY-16379), baricitinib (HY-15315), tofacitinib (HY-40354), CI-1040 (HY-50295), TPL2 kinase inhibitor 1 (HY-12358), and COT inhibitor 2 (HY-32018) were purchased from MedChemExpress. For in vitro experiments, each drug was diluted in DMSO, and doses were determined using viability assays. To evaluate the effect of ruxolitinib on cSCC proliferation, we used a large panel of doses ranging from 0.097 to 12.5 μM. For further in vitro mechanistic and signaling studies, we used 3.125 μM, as this dose consistently increased cSCC proliferation across 2 different readouts (Cell-Titer Glo luminescence in Figure 2A and Figure 4G, and live-cell imaging in Figure 2B). We confirmed that this dose efficiently impaired JAK/STAT signaling, resulting in decreased phosphorylation of both STAT3 and STAT5 by Western blot, as shown in Figure 3E. Drugs were changed every 48 hours.

Calyculin A, a serine/threonine phosphatase inhibitor (PP1 and PP2A), was purchased from Sigma-Aldrich (C5552), and cells were treated for 30 min at the indicated concentrations.

#### Viability assay.

To assess cell viability and determine the dose of drugs to use for each cell line, cells were plated in 384-well cell culture–coated white plates. To measure ATP content, CellTiter-Glo reagent (G7573, Promega) was added to each well and incubated at room temperature for 25 min prior to luminescence reading at the indicated time points.

#### Proliferation assay.

For proliferation assays, cells were imaged every 24 hours in whole-well condition by an IncuCyte S3 Live-Cell Analysis instrument (Sartorius). Cell proliferation was determined as percent confluence from phase images or as count number/surface area from fluorescence images. Images were analyzed using IncuCyte image analysis software (Sartorius).

### Cloning, cell transfection, and transduction

#### Cloning.

shRNA constructs targeting *COT* (*MAP3K8*), *JAK1*, and *JAK2* (sequences listed in Supplemental Table 12) were cloned into the SGEN GFP^+^ vector (Addgene, 111171) and the LT3REVIR GFP^+^DsRed^+^ vector (Addgene, 111176) for COT and into the LT3REVIR GFP^+^DsRed^+^ vector (Addgene, 111176) for JAK1 and JAK2 as XhoI-EcoRI fragments, which were generated by amplifying 97-mer oligonucleotides (Integrated DNA Technologies) using 5′miRE-XhoI (TGAACTCGAGAAGGTATATTGCTGTTGACAGTGAGCG) and 3′miRE-EcoRI (TCTCGAATTCTAGCCCCTTGAAGTCCGAGGCAGTAGGC) primers. Each PCR reaction followed these conditions: 50 μL reaction containing 1 μM oligonucleotide template, 5 μM of each primer, 25 μL of Phusion master mix (M0536L, New England Biolabs), 0.75 μL of DMSO, and 18.25 μL of RNase-free water; cycling: 98°C for 30 s; 35 cycles of 98°C for 30 s, 54°C for 30 s, and 72°C for 20 s; and 72°C for 5 min.

Codon-optimized synthetic double-stranded DNA fragments encoding the WT and T290A mutant forms of COT were synthesized by Integrated DNA Technologies. PCR products generated from amplification of each fragment were cloned into the pLVX eBFP-puro vector (54, 55) via double digestion with BamHI and EcoRI restriction enzymes. The sequences of positive clones were confirmed by Sanger sequencing.

#### Transfection.

For virus production, 18 × 10^6^ HEK293T cells were transfected with 13.5 μg DNA for each lentiviral vector and with 11 μg of PAX2 and 5.5 μg of VSVG packaging vectors. Viral supernatants were harvested 72 hours later and filtered using 0.45 μm filters.

#### Transduction.

Lentiviral supernatant, 25 mM HEPES (Sigma-Aldrich), and 8 μg/mL polybrene (Sigma-Aldrich) were added to the target cells. In cells transduced with LT3R backbone, shRNA expression was induced by doxycycline addition at 1 μM. Transduced cells were selected 24–72 hours later by sorting according to the expressed fluorescent marker. Cells transduced with pLVX eBFP-puro backbone were selected by puromycin addition at 1 μg/mL.

### RT-qPCR analysis

RNA was extracted using the RNeasy Mini Kit (Qiagen), and reverse transcription was performed using SuperScript IV reverse transcriptase (Invitrogen) per the manufacturer’s protocols. Real-time qPCR was performed using the TaqMan protocol (Applied Biosystems). For quantification of h*MAP3K8* (h*COT*), h*JAK1*, h*JAK2*, and h*GAPDH*, the following TaqMan probes were used: Hs01566396_m1, Hs01026983_m1, and Hs01078136_m1 probes conjugated to FAM-MGB and Hs02786624_g1 probe conjugated to VIC-MGB. Data was analyzed using the QuantStudio real-time PCR instrument and software (Thermo Fisher Scientific).

### Western blot

Cells were lysed in Cell Signaling Lysis Buffer (Cell Signaling Technology) and quantified using protein assay dye reagent (Bio-Rad). Protein migration was performed by gel electrophoresis using 4%–12% Bis-Tris gels (Sigma-Aldrich). Proteins were next transferred to PVDF membranes (Merck) and blocked for 1 hour in 3% BSA (Sigma-Aldrich). Blots were incubated with primary antibodies (1:1,000) overnight at 4°C and washed 3 times, followed by incubation with secondary antibodies (1:5,000) for 1 hour at room temperature. Total protein signal was detected using Pierce ECL Western Blotting Substrate (Thermo Fisher Scientific) and phospho-protein signal using SuperSignal West Pico PLUS Chemiluminescent Substrate (Thermo Fisher Scientific).

The antibodies used for Western blotting are listed in Supplemental Table 13.

### In vivo experiments: chemically induced cSCC murine model

#### Three-step model.

This model, described by Abel et al. (56), comprises 3 phases: (a) initiation, in which an initiating oncogenic agent is applied to shaved skin of mice to initiate cutaneous carcinogenesis; (b) promotion, in which a proinflammatory agent is applied to shaved skin of mice twice weekly, leading to epidermal hyperplasia and precancerous lesions, named papillomas; and (c) progression, in which a subset of papillomas transform into overt cSCC.

#### Mice.

7-week-old female FVB/N mice (Janvier Labs) were used.

#### Initiating and promoting agents.

200 nM 7,12-dimethylbenz(a)-anthracene (DMBA; D3254, Sigma-Aldrich) and 5 nM 12-*O*-tetradecanoylphorbol-13-acetate (TPA; P-1680, LC Laboratories) in 0.2 mL of acetone solution (0.2 μL per mouse) were applied to the shaved back skin of the mice at weeks 0 and 3 and twice weekly from weeks 4 to 27.

#### Clinical evaluation.

Papilloma and cSCC number and volume were evaluated weekly.

#### Drug administration.

Ruxolitinib (HY-50856, MedChemExpress) was diluted in a solvent composed of 5% *N*,*N*-dimethylacetamide (8032351000, Merck Millipore) and 0.5% methocellulose (43147, Thermo Fisher Scientific) diluted in water. Ruxolitinib was used at 45 mg/kg bid by gavage during 2 consecutive weeks followed by 3 weeks of interruption and then restarted 1 week prior to the end of the experiment. This dose was chosen based on the following findings. The dose of ruxolitinib used in the initial preclinical validation publication of ruxolitinib efficacy and pharmacodynamics was 90 mg/kg bid in BALB/c mice (57). A more recent study used a lower dose (60 mg/kg bid) in C57BL/6J mice (58). Since our skin cancer model was developed in FvB/N mice and because no data were available in the literature for this model and mice strain, we evaluated the tolerance of 2 different doses: 90 and 45 mg/kg bid. The 90 mg/kg bid regimen was not well tolerated, with 3 deaths among 9 mice treated. The 45 mg/kg bid dose did not induce any toxic death in 7 mice treated and was well tolerated during the treatment duration (no weight loss compared with vehicle control–treated mice; Supplemental Figure 3A). Importantly, we validated that this dose of ruxolitinib is sufficient to observe a pharmacodynamic effect on the JAK/STAT pathway, as shown by reduction of P-STAT5 and P-STAT3 expression in skin tumors obtained from mice treated by ruxolitinib (Supplemental Figure 3B). JAK/STAT signaling downregulation in ruxolitinib-mediated tumors was also validated at the transcriptional level, as shown by GSEA in Figure 3A.

CI-1040 (HY-50295, MedChemExpress) was diluted in a solvent composed of 10% DMSO and 90% water. CI-1040 was used at 25 mg/kg once daily by gavage during 6 consecutive weeks.

### Melanoma murine models

500,000 melanoma murine cells (B16-OVA and YUMMER1.7) were injected into the right side of 7-week-old C57BL/6 mice purchased from Envigo. At tumor detection (4 days after injection), ruxolitinib (HY-50856, MedChemExpress) was used at 90 mg/kg bid by gavage for 10 consecutive days. Ruxolitinib was diluted in a solvent composed of 5% *N*,*N*-dimethylacetamide and 0.5% methocellulose diluted in water.

### Histological and immunofluorescence analysis of murine samples

The methodology is detailed in Supplemental Methods.

### Cell cycle analysis

The A431 cSCC cell line was harvested at day 3 after treatment initiation, washed in PBS, and fixed in ice-cold 70% ethanol for a minimum of 12 hours. Cells were then washed in PBS, resuspended in PBS, and incubated for 5 min at 4°C with RNase A (30 mg/mL, Sigma-Aldrich), prior to their incubation for 30 min in propidium iodide (1 mg/mL, Invitrogen). FACS analysis was performed on a BD FACSCanto II instrument (BD Biosciences) directly after incubation. Data were analyzed using FlowJo v10.0.1 (Becton, Dickinson and Company).

### Phosphoproteomic analysis and RNA sequencing

The methodology is detailed in Supplemental Methods.

### Statistics

Continuous variables were reported as medians and IQRs, while categorical variables were reported as numbers and proportions. The primary endpoint of the study was cumulative incidence of cSCC, defined from the date of MPN diagnosis until the date of cSCC diagnosis, censoring patients alive without cSCC at last follow-up. Univariable and multivariable analyses assessing the impact of categorical and continuous variables on cSCC development during follow-up were performed using a Fine-Gray model. Death without cSCC was considered a competitive event. Age, MPN subtype, sex, and treatment with a JAK inhibitor were included in the univariable analysis. Treatment with JAK inhibitor was considered a time-dependent covariate. The proportional hazard assumption was validated by testing Schoenfeld residuals. All significant variables (*P* < 0.05) were included in the multivariable analysis, performed by the Fine-Gray model followed by backward stepwise selection. HRs are given with 95% CI. Statistical analyses were performed using STATA 15.0 software (StataCorp).

Statistical analysis for in vitro and in vivo experiments was performed using Prism 8.0.1 (GraphPad). Pairwise comparisons between 2 independent groups were performed using the Mann-Whitney *U* test. Comparisons involving multiple groups were analyzed using 1- or 2-way ANOVA, as appropriate, according to the number of experimental factors, followed by Tukey’s multiple-comparison test. All tests were 2 tailed, and a *P* value of less than 0.05 was considered significant. Parameters such as number of replicates, measures of dispersion (mean ± SD or SEM), statistical test, and significance are provided in the figures and figure legends.

### Study approval

The human study was performed in accordance with the ethical guidelines of the Declaration of Helsinki and was approved by the Groupe Hospitalo-Universitaire AP-HP (Assistance Publique–Hôpitaux de Paris) Nord institutional review board (IRB00006477, CER-2020-55). The French National Ethics Committee on Animal Care reviewed and approved all mouse experiments described in this study (authorization numbers APAFIS 23169-2019120416018976 v4 and 28821-20210104093490 v4). Mice were euthanized using cervical dislocation if animals showed limit points (weight loss > 20% or signs of major tumor progression).

### Data availability

RNA-seq data from control and ruxolitinib cSCC mice tumors are available in the Gene Expression Omnibus database under accession number GSE278530. All other data from this study are available in the paper, the supplementary materials, or in the Supplemental Data Values file. Further information is available from the corresponding author upon reasonable request.

## Manuscript information

### Author contributions.

HP: conceptualization, formal analysis, validation, investigation, visualization, methodology, and writing (original draft). AF: conceptualization, formal analysis, visualization, and methodology. MB: conceptualization, formal analysis, investigation, visualization, and methodology. RM and LP: investigation, validation, and methodology. BB: conceptualization, resources, and methodology. EL, KS, and JL: formal analysis and visualization. CC, BR, DL, FJ, and BL: investigation and methodology. AG, MD, and PN: conceptualization, formal analysis, and visualization. STD, LA, and ER: resources. BC: investigation, formal analysis, and methodology. JSD, JJK, and C Lebbé: conceptualization and resources. SM: conceptualization, investigation, resources, and methodology. C Lobry: conceptualization, investigation, formal analysis, and visualization. AP: conceptualization, investigation, methodology, and resources. LB: conceptualization, formal analysis, supervision, funding acquisition, investigation, methodology, writing (original draft), and project administration.

### AI disclosure.

The authors declare that no AI tools were used in the original research for or writing of this article.

### Conflict of interest.

LB has received research support from Gilead and Pfizer and consulted for Novartis, GSK, Menarini Stemline, and Takeda. MB has received research support from Takeda and Kyowa Kirin and consulted for Bristol-Myers Squibb, Innate Pharma, Kyowa Kirin, Takeda, and Cerba Research.

### Funding.

This study was supported by the following:

- ERC Starting program (101117339; to LB).
- Ligue Nationale Contre le Cancer and ATIP-Avenir 2022 program (23AA019-00; to LB).
- Fondation Bettencourt-Schueller (CCA-INSERM-Bettencourt program 2019; to LB).
- Société Française de Dermatologie (AO2021; to LB).
- Emergence Ville de Paris (2021 DAE 100; to LB).
- SIRIC InsiTu program (INCa-DGOS-INSERM-ITMO Cancer_18008; to LB, C Lobry, and AP).
- IHU France 2030 “Leukemia Institute Paris Saint-Louis” (ANR-23-IAHU-0005; to LB, C Lobry, and AP).
- INSERM ITMO Cancer FRFT-Doc scholarship (to HP).
- CCA-INSERM-Bettencourt program (2025) (to HP).

## Supplementary Material

Supplemental data

Unedited blot and gel images

Supplemental table 8

Supplemental table 9

Supporting data values

## Figures and Tables

**Figure 1:**
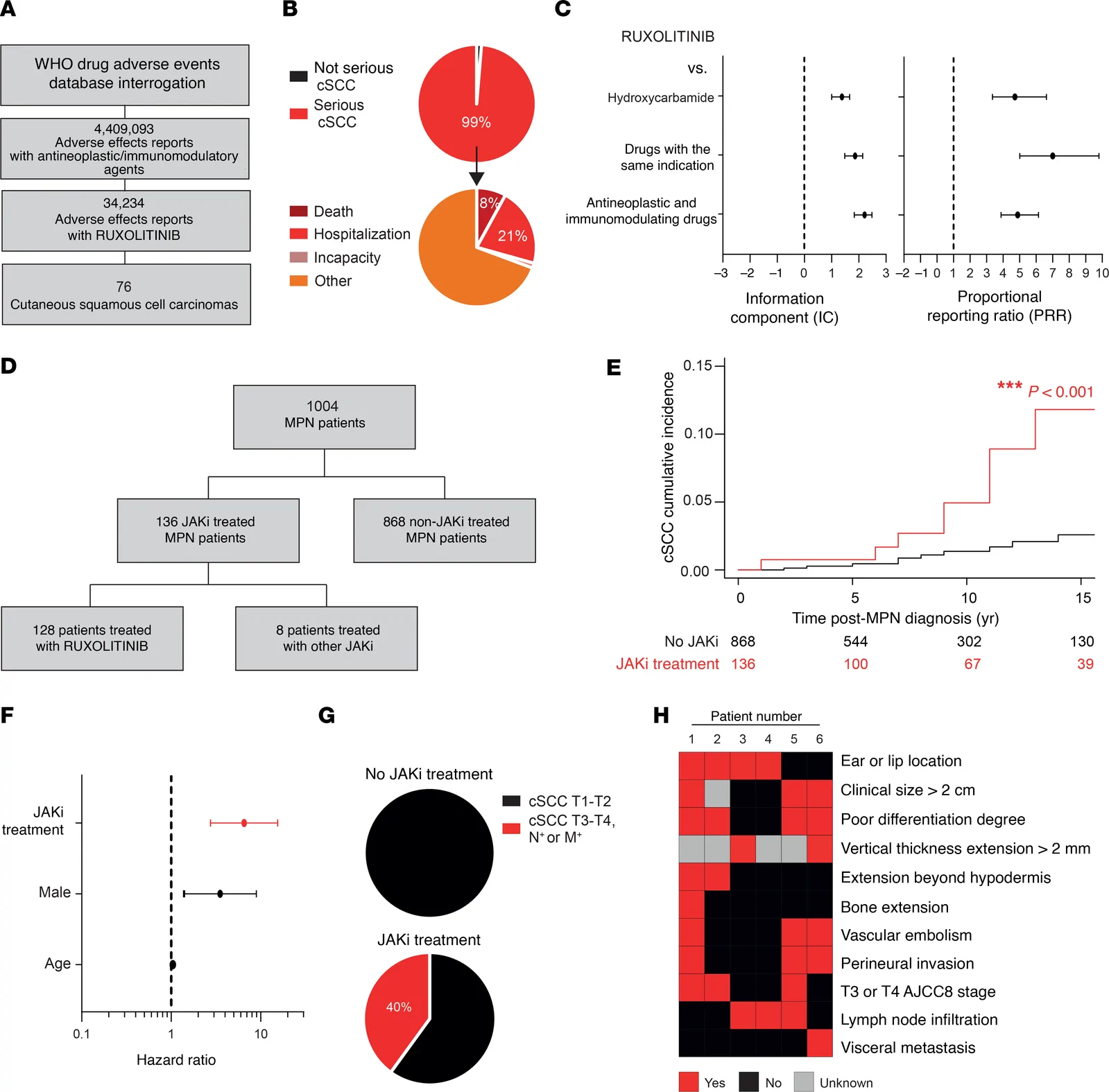
Ruxolitinib exposure is associated with the development of aggressive cSCC. (**A**) Flowchart depicting the cases included in the international pharmacovigilance study. (**B**) Pie chart depicting the severity of cSCC reported under ruxolitinib treatment according to the international pharmacovigilance database (VigiBase). (**C**) IC and PRR measuring the association between cSCC and ruxolitinib versus the indicated comparators. (**D**) Flowchart depicting patients diagnosed with MPNs included in the study according to their exposure to ruxolitinib and other JAK inhibitors (JAKi). (**E**) Cumulative incidence of cSCC for JAK inhibitor–treated patients compared with non–JAK inhibitor–treated patients. The Fine-Gray model was used for group comparison. ***P < 0.001. (**F**) Forest plot depicting factors independently associated with cSCC development in multivariate Fine-Gray analysis. (**G**) Proportion of localized cSCC (in black) and locally advanced (T3–T4) or metastatic (N^+^ or M^+^) cSCC (in red) among MPN patients treated with JAK inhibitor or not. (**H**) Heatmap depicting the clinical and histological aggressiveness criteria of the 6 aggressive cSCCs developed under JAK inhibitor treatment in patients with MPN. Presence or absence of aggressive criteria is represented in red or black, respectively. Absence of information is represented in gray. TNM status was defined according to AJCC8 classification.

**Figure 2:**
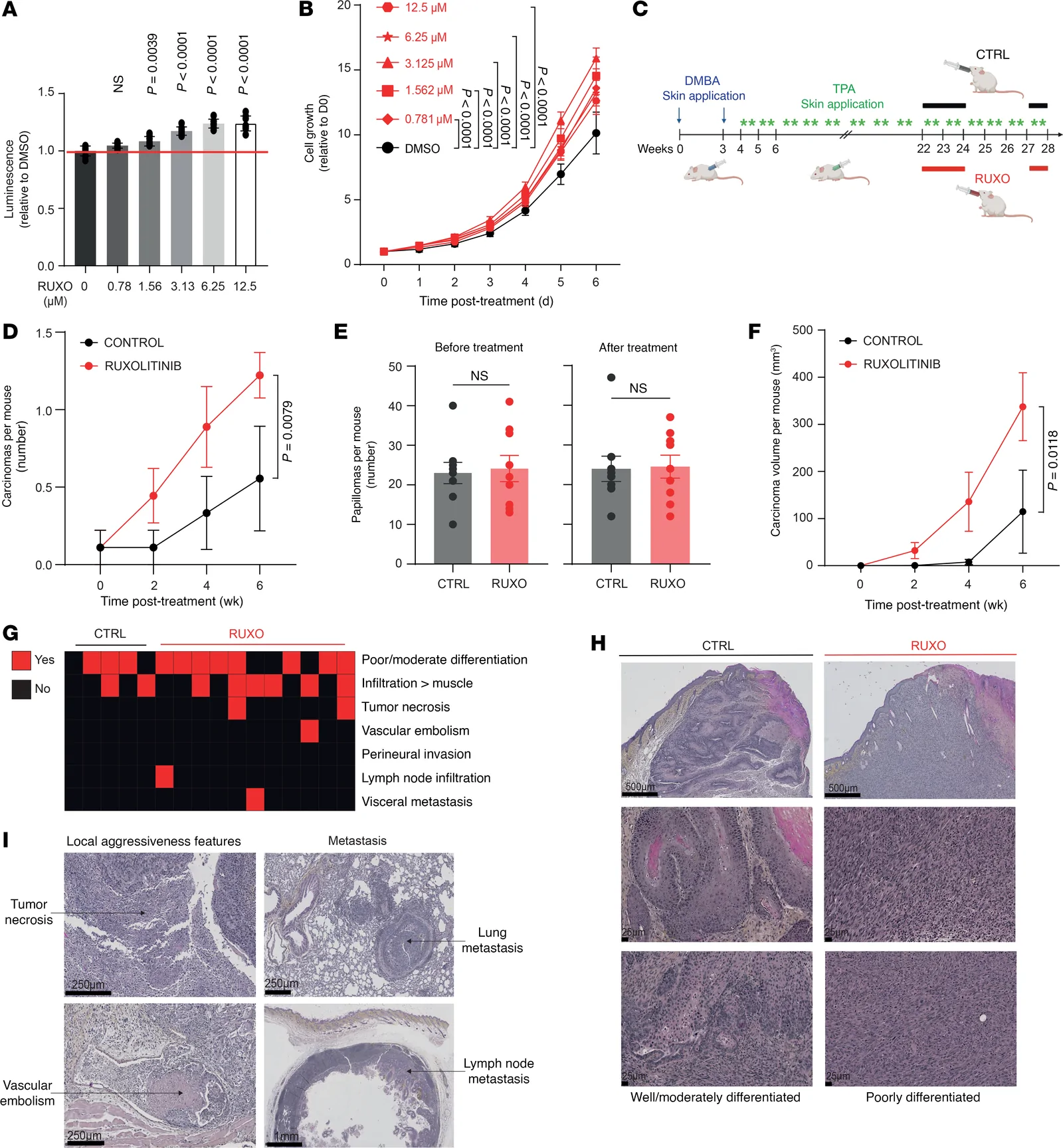
Ruxolitinib treatment promotes aggressive cSCC in vitro and in vivo. (**A**) Cell growth of A431 cSCC cell line treated with increasing doses of ruxolitinib for 3 days. Data represent mean ± SD of 8 replicates. Statistical significance was determined using 1-way ANOVA. Conditions above the red horizontal line indicate increased proliferation under ruxolitinib compared with DMSO. This experiment was performed 2 times. (**B**) Cell growth of A431 human cSCC cell line treated with increasing doses of ruxolitinib. Statistical significance was determined using 2-way ANOVA at day 6. Data represent mean ± SD of 8 replicates. This experiment was performed 2 times. (**C**) Schematic of the chemically induced murine cSCC model. DMBA was applied at weeks 0 and 3 to initiate cSCC carcinogenesis, followed by TPA twice weekly from week 4 to induce hyperplasia and premalignant lesions (papillomas). A subset of papillomas transformed into cSCC during the progression phase. At 22 weeks, mice (*n* = 9 per group) received vehicle or ruxolitinib (45 mg/kg bid) for 2 weeks. After 3 weeks of treatment interruption, treatment was resumed 1 week before endpoint. Created in BioRender. (**D**) Cutaneous carcinoma number per mouse in ruxolitinib- versus vehicle-treated mice. Statistical significance was determined using Mann-Whitney test. Data represent mean ± SEM of 9 mice. (**E**) Papilloma number per mouse under ruxolitinib treatment compared with control mice, before (left) and after treatment initiation (right). Statistical significance was determined using Mann-Whitney test. Data represent mean ± SEM of 9 mice. (**F**) Cutaneous carcinoma volume in ruxolitinib- versus vehicle-treated mice. Statistical significance was determined using Mann-Whitney test. Data represent mean ± SEM of 9 mice. (**G**) Heatmap depicting histological aggressiveness criteria of cSCC from ruxolitinib- versus vehicle-treated mice. Presence or absence of aggressiveness criteria is represented in red or black, respectively. (**H**) Representative H&E micrographs of 3 tumors per group comparing tumor differentiation degree. Scale bars: 500 μm (top), 25 μm (middle and bottom). (**I**) Representative H&E micrographs showing cSCC histological aggressiveness criteria in ruxolitinib-associated mice tumors. Scale bars: 250 μm (top and bottom left), 1 mm (bottom right).

**Figure 3:**
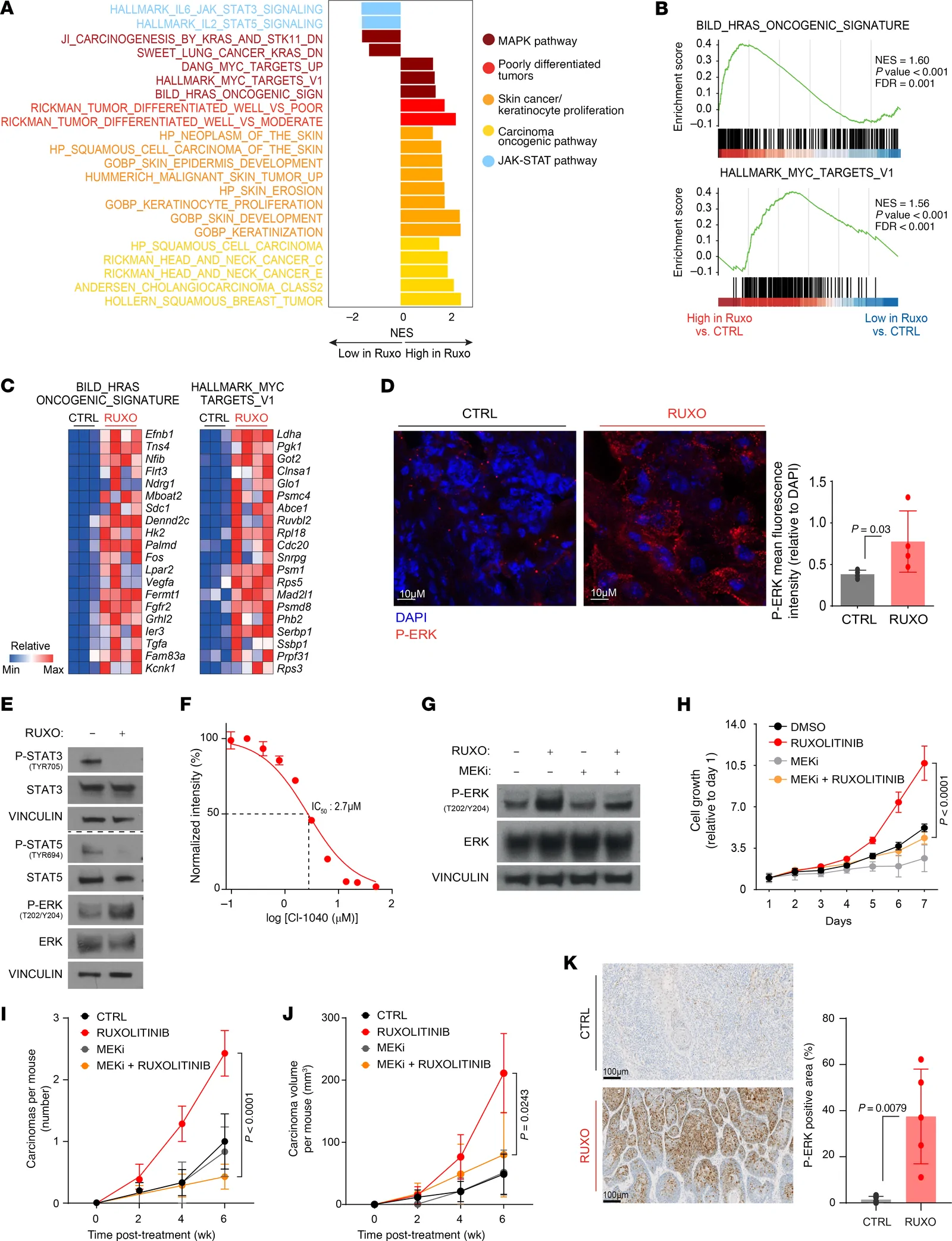
MAPK pathway activation drives ruxolitinib-associated cutaneous carcinogenesis. (**A**) GSEA of RNA-seq data identifying upregulated oncogenic pathways in cutaneous carcinomas from ruxolitinib-treated (*n* = 4) versus control mice (*n* = 3). Gene sets with FDR < 0.25 were considered enriched. NES, normalized enrichment score. (**B**) Representative GSEA plots showing enrichment of MAPK-related gene sets in ruxolitinib-associated tumors. (**C**) Heatmaps of the top 20 leading-edge genes from 2 MAPK-related gene sets comparing expression from ruxolitinib (RUXO; *n* = 4) and control (CTRL; *n* = 3) mice tumors. Blue and red indicate decreased and increased expression, respectively. (**D**) Representative immunofluorescence images of phosphorylated ERK (P-ERK; red) in tumors from ruxolitinib- (*n* = 4) and vehicle-treated mice (*n* = 4), with quantification of P-ERK fluorescence intensity relative to DAPI. Statistical significance was determined using Mann-Whitney test. Data represent mean ± SD of 4 mice. Scale bars: 10 μm. (**E**) Phosphorylation level of ERK, STAT3, and STAT5 by Western blot in A431 cSCC cell line treated with ruxolitinib (3.125 μM) compared with DMSO. (**F**) Growth inhibition curve and IC_50_ value of A431 cSCC cell line in response to increasing doses of CI-1040 after 3 days of treatment. Data represent mean ± SD of 8 replicates. This experiment was performed 2 times. (**G**) Phosphorylation level of ERK by Western blot in A431 cSCC cell line treated with DMSO, ruxolitinib (3.125 μM), CI-1040 (3 μM), or CI-1040 and ruxolitinib. (**H**) Cell growth of A431 cSCC human cell line under DMSO (in black), ruxolitinib (in red, 3.125 μM), CI-1040 (MEKi in gray, 3 μM), or both ruxolitinib and CI-1040 (in orange) treatments. Statistical significance was determined using 2-way ANOVA at day 7. Data represent mean ± SD of 8 replicates. This experiment was performed 2 times. (**I** and **J**) Cutaneous carcinoma number per mouse (**I**) and volume (**J**) in mice treated with ruxolitinib (in red, 45 mg/kg bid, *n* = 7), CI-1040 (MEKi in gray, 25 mg/kg qd, *n* = 6), or both drugs (in orange, *n* = 7) compared with vehicle-treated control mice (in black, *n* = 6). Statistical significance was determined using 2-way ANOVA at week 6 after treatment. Data represent mean ± SEM of 6 (vehicle and MEKi groups) or 7 (ruxolitinib and ruxolitinib + MEKi groups) mice. (**K**) Representative immunochemistry images (left) depicting ERK phosphorylation level in aggressive cSCC developed in ruxolitinib-treated MPN patients (*n* = 5) compared with matched spontaneously developed cSCC (*n* = 5), with quantification of P-ERK^+^ area. Statistical significance was determined using Mann-Whitney test. Data represent mean ± SD of 5 tumors. Scale bars: 100 μm.

**Figure 4:**
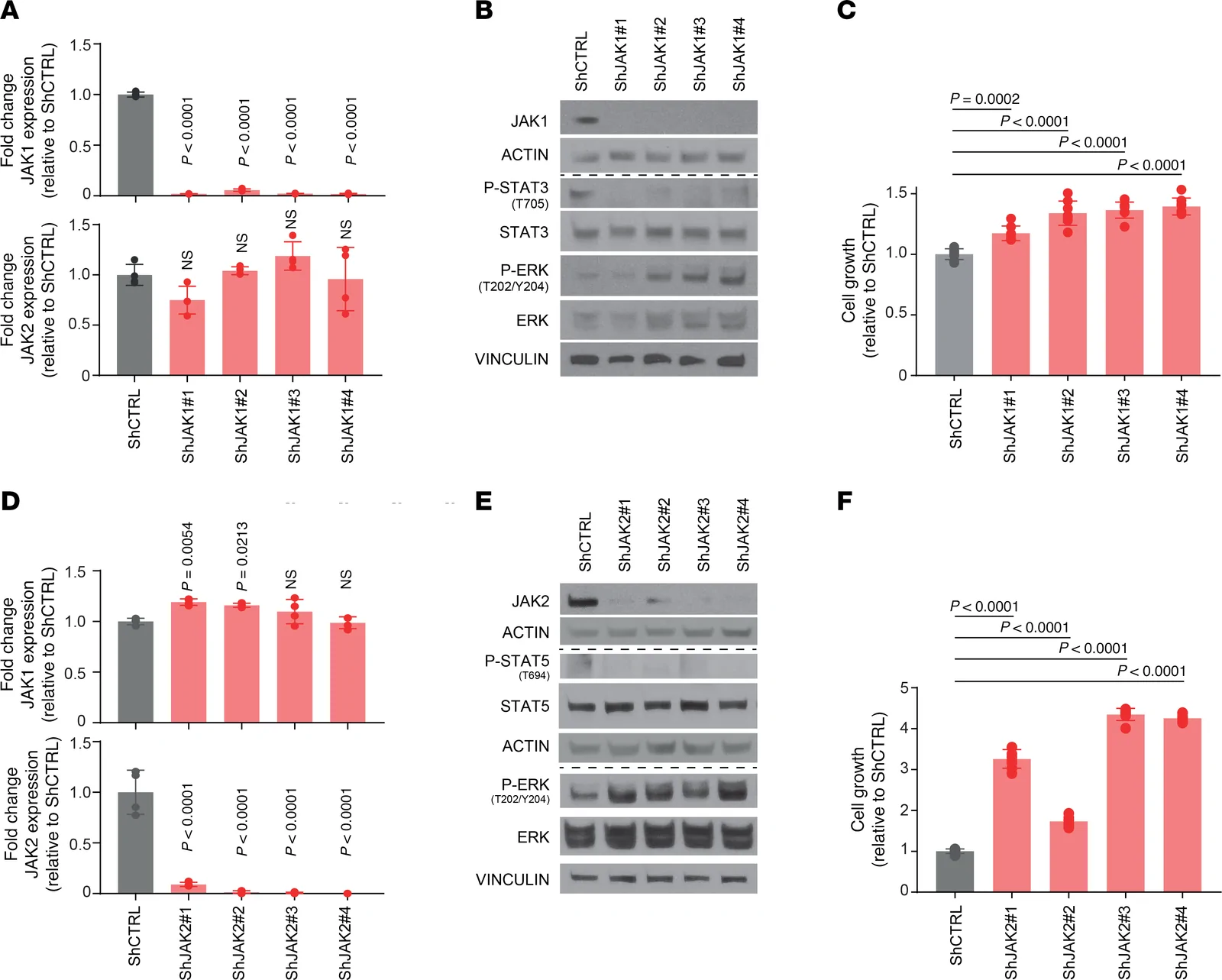
Ruxolitinib-associated cutaneous carcinogenesis results mostly from JAK2 inhibition. (**A**) RT-qPCR for *JAK1* (top) and *JAK2* (bottom) expression level in A431 cSCC cell line transduced with either a nontargeting control shRNA or *JAK1*-directed shRNAs (*n* = 4). Statistical significance was determined using 1-way ANOVA. Data represent mean ± SD of 4 replicates. This experiment was performed 2 times. (**B**) Level of JAK1, STAT3, P-STAT3, ERK, P-ERK, ACTIN, and VINCULIN by Western blot in A431 cSCC cell line transduced with either a nontargeting control shRNA or *JAK1*-directed shRNAs (*n* = 4) after 2 days of doxycycline-induced shRNA expression. (**C**) Cell growth of A431 cSCC cell line transduced with either a nontargeting control shRNA (shCTRL) or *JAK1*-directed shRNAs (*n* = 4) after 4 days of doxycycline-induced shRNA expression. Statistical significance was determined using 1-way ANOVA. Data represent mean ± SD of 8 replicates. This experiment was performed 2 times. (**D**) RT-qPCR for *JAK1* (top) and *JAK2* (bottom) expression level in A431 cSCC cell line transduced with either a nontargeting control shRNA or *JAK2*-directed shRNAs (*n* = 4). Statistical significance was determined using 1-way ANOVA. Data represent mean ± SD of 4 replicates. This experiment was performed 2 times. (**E**) Level of JAK2, STAT5, P-STAT5, ERK, P-ERK, ACTIN, and VINCULIN by Western blot in A431 cSCC cell line transduced with either a nontargeting control shRNA or *JAK2*-directed shRNAs (*n* = 4) after 2 days of doxycycline-induced shRNA expression. (**F**) Cell growth of A431 cSCC cell line transduced with either a nontargeting control shRNA (shCTRL) or *JAK2*-directed shRNAs (*n* = 4) after 4 days of doxycycline-induced shRNA expression. Statistical significance was determined using 1-way ANOVA. Data represent mean ± SD of 8 replicates. This experiment was performed 2 times.

**Figure 5:**
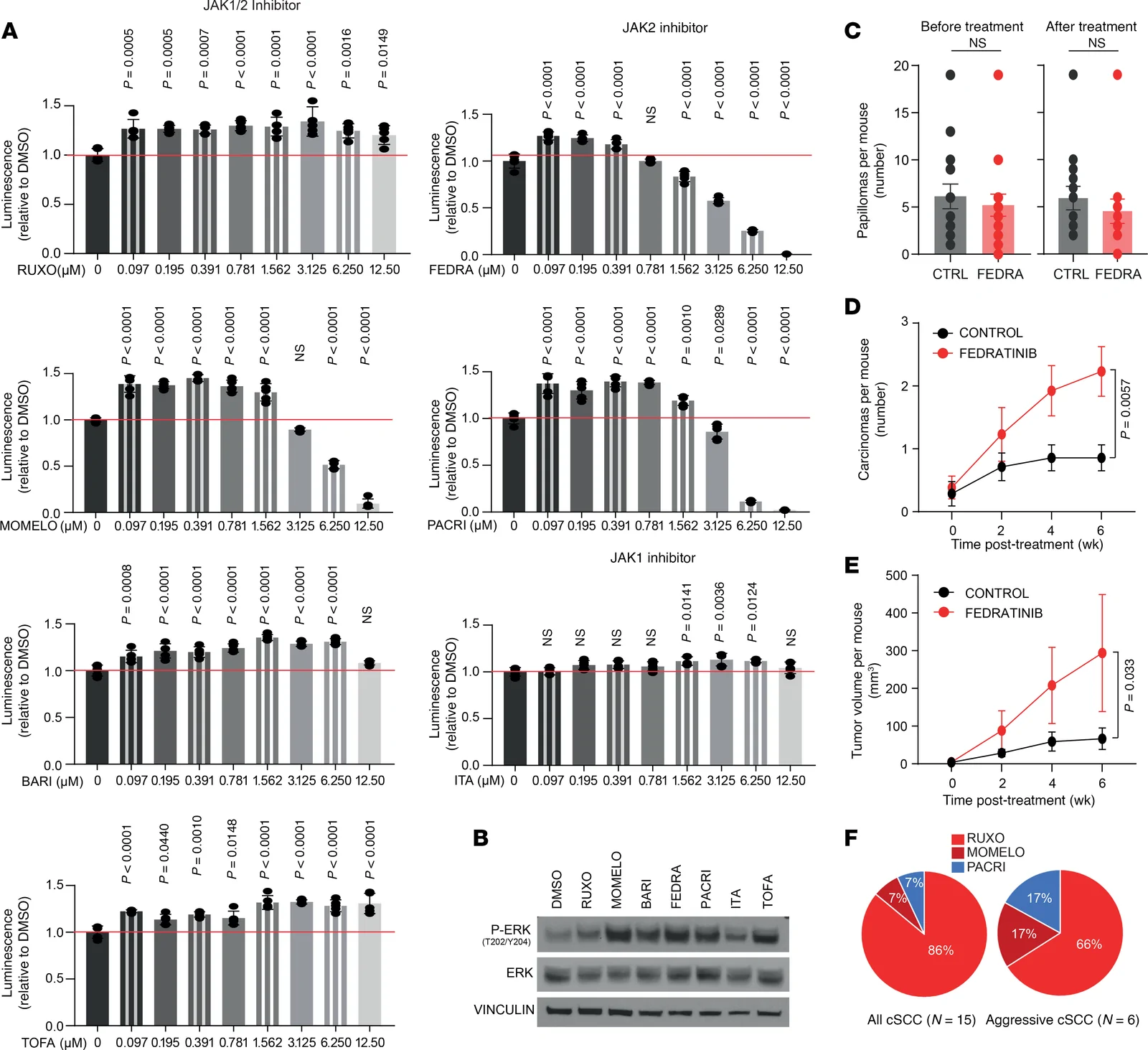
cSCC promotion is a JAK inhibitor–associated class effect. (**A**) Cell growth of A431 cSCC cell line treated with increasing doses of JAK1/2 inhibitors (ruxolitinib, momelotinib, baricitinib, and tofacitinib), JAK2-specific inhibitors (fedratinib and pacritinib), or JAK1-specific inhibitor (itacitinib) for 3 days. Data represent mean ± SD of 5 replicates. This experiment was performed 2 times. Statistical significance was determined using 1-way ANOVA. Conditions above the red horizontal line indicate increased proliferation under JAK inhibitor treatment compared with DMSO. (**B**) Phosphorylation level of ERK by Western blot in A431 cSCC cell line treated with JAK1/2 inhibitors (ruxolitinib, 3.125 μM; momelotinib, 0.391 μM; baricitinib, 1.562 μM; tofacitinib, 3.125 μM), JAK2-specific inhibitors (fedratinib, 0.097 μM; pacritinib, 0.391 μM), or JAK1-specific inhibitor (itacitinib, 3.125 μM). (**C**) Papilloma number per mouse under fedratinib (*n* = 15) treatment compared with control mice (*n* = 15), before (left) and after treatment initiation (right). Statistical significance was determined using Mann-Whitney test. Data represent mean ± SEM of 14 (vehicle group) or 13 (fedratinib group) mice. (**D**) Cutaneous carcinoma number per mouse in mice treated with fedratinib (*n* = 15) compared with vehicle-treated control mice (*n* = 15). Statistical significance was determined using Mann-Whitney test. Data represent mean ± SEM. (**E**) Cutaneous carcinoma volume in mice treated with fedratinib (*n* = 15) compared with vehicle-treated control mice (*n* = 15). Statistical significance was determined using Mann-Whitney test. Data represent mean ± SEM. (**F**) Pie chart reporting the proportion of all cSCCs (left) and aggressive cSCCs (right) under different JAK inhibitors in a cohort of 1,004 patients with MPN. RUXO, ruxolitinib; MOMELO, momelotinib; PACRI, pacritinib.

**Figure 6:**
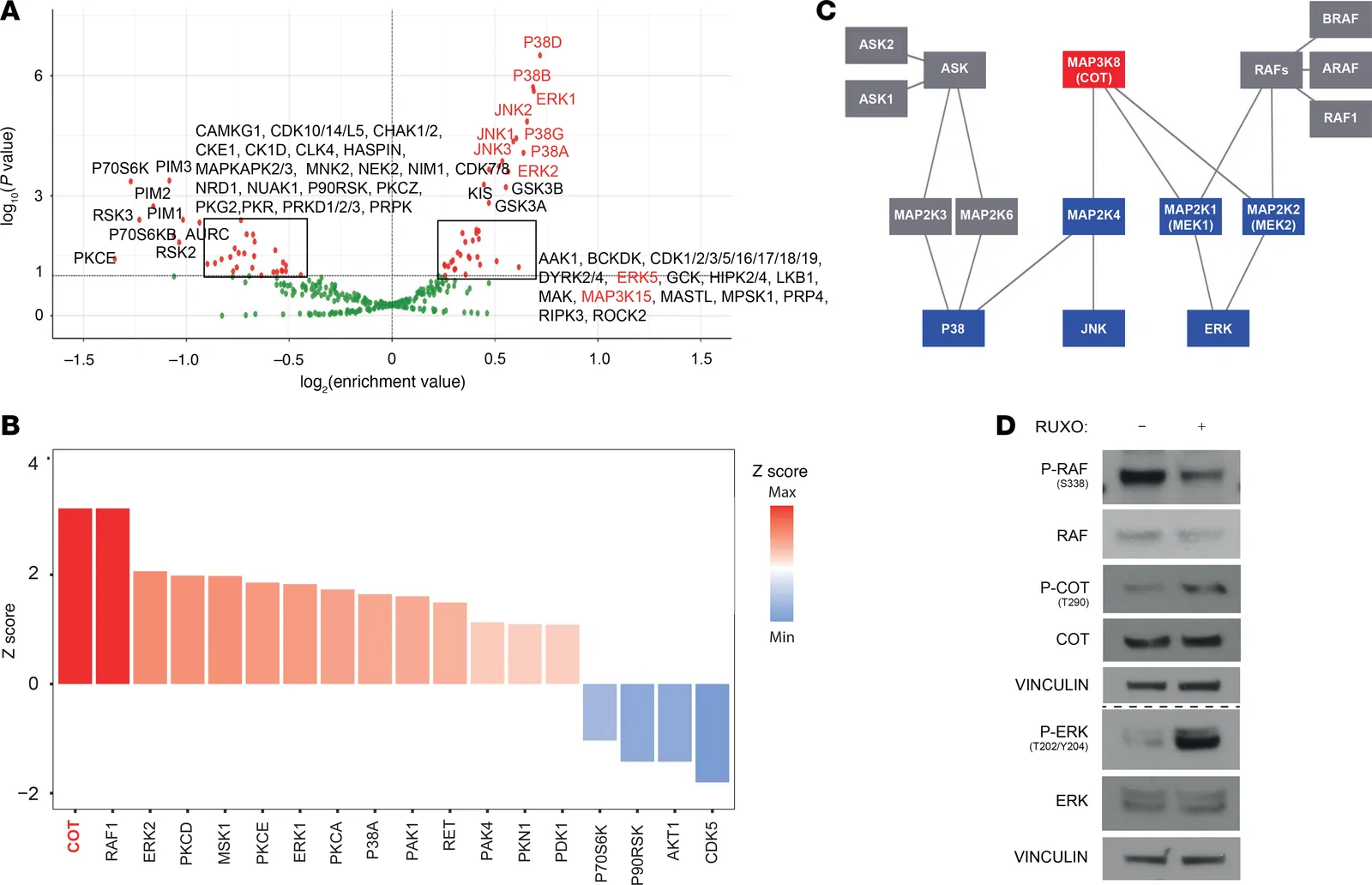
Ruxolitinib-induced MAPK pathway activation is associated with COT kinase activation. (**A**) KEA in cutaneous carcinomas developed by ruxolitinib-treated mice (*n* = 5) compared with control mice (*n* = 4). Kinases are considered enriched if log_2_ fold change > 1 and *P* value < 0.05. MAPKs significantly enriched are highlighted in red. (**B**) Robust kinase activity inference KEA in cutaneous carcinomas developed by ruxolitinib-treated mice (*n* = 5) compared with control mice (*n* = 4). (**C**) Network of COT kinase substrates within the MAPK pathway. COT is highlighted in red, and its MAPK downstream signaling is in blue. Other MAPK-activating proteins are in gray. (**D**) Phosphorylation level of RAF, COT, and ERK by Western blot in A431 cSCC cell line treated with ruxolitinib (3.125 μM) compared with DMSO.

**Figure 7:**
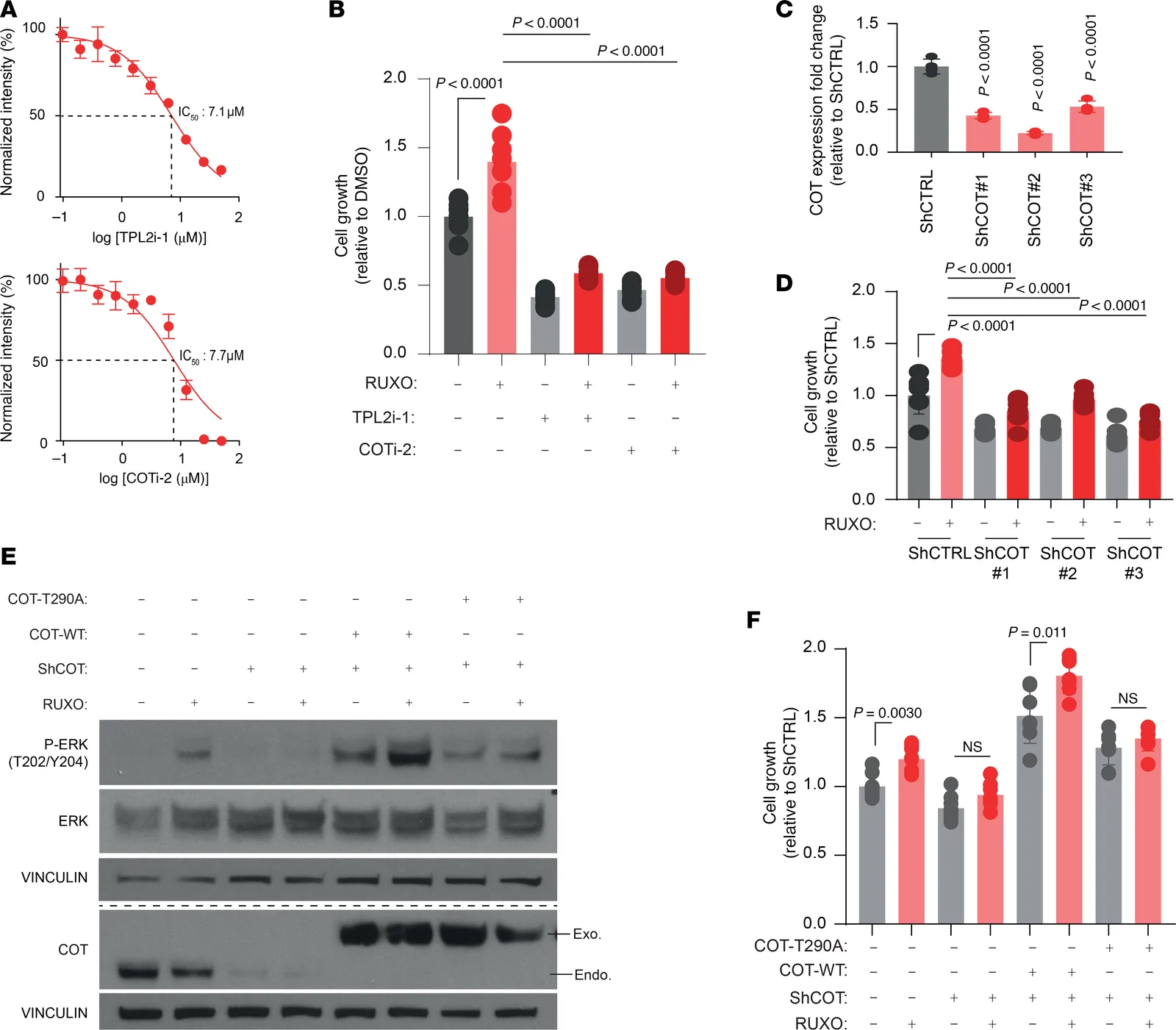
COT kinase phosphorylation mediates ruxolitinib-induced cutaneous carcinogenesis. (**A**) Growth inhibition curve and IC_50_ value of A431 cSCC cell line in response to increasing doses of TPL2i-1 and COTi-2 after 3 days of treatment. Data represent mean ± SD of 8 replicates. This experiment was performed 2 times. (**B**) Cell growth of A431 cSCC cell line after 6 days of treatment with DMSO (in black), ruxolitinib (in red, 3.125 μM), COT inhibitors (in gray, TPL2i-1 or COTi-2 at 7 μM), or ruxolitinib and COT inhibitors (in dark red). Statistical significance was determined using 1-way ANOVA. Data represent mean ± SD of 8 replicates. This experiment was performed 2 times. (**C**) RT-qPCR for COT expression level in A431 cSCC cell line transduced with either a nontargeting control shRNA or COT-directed shRNAs (*n* = 3). Statistical significance was determined using 1-way ANOVA. Data represent mean ± SD of 4 replicates. (**D**) Cell growth of A431 cSCC cell line transduced with either a nontargeting control shRNA (shCTRL) or COT-directed shRNAs (*n* = 3) after 4 days of DMSO or ruxolitinib treatment (3.125 μM). Statistical significance was determined using 1-way ANOVA. Data represent mean ± SD of 8 replicates. This experiment was performed 2 times. (**E**) ERK phosphorylation and COT expression levels by Western blot in A431 cSCC cell line transduced with (a) either a nontargeting control shRNA (shCTRL) or COT-directed shRNA and (b) either a codon-optimized WT COT construct or a T290A-COT mutant and treated with ruxolitinib (3.125 μM) compared with DMSO. (**F**) Cell growth of A431 cSCC cell line transduced with (a) either a nontargeting control shRNA (shCTRL) or COT-directed shRNA and (b) either a WT COT construct or a T290A-COT mutant after 4 days of DMSO or ruxolitinib treatment (3.125 μM). Statistical significance was determined using Mann-Whitney test. Data represent mean ± SD of 8 replicates. This experiment was performed 2 times.

**Figure 8:**
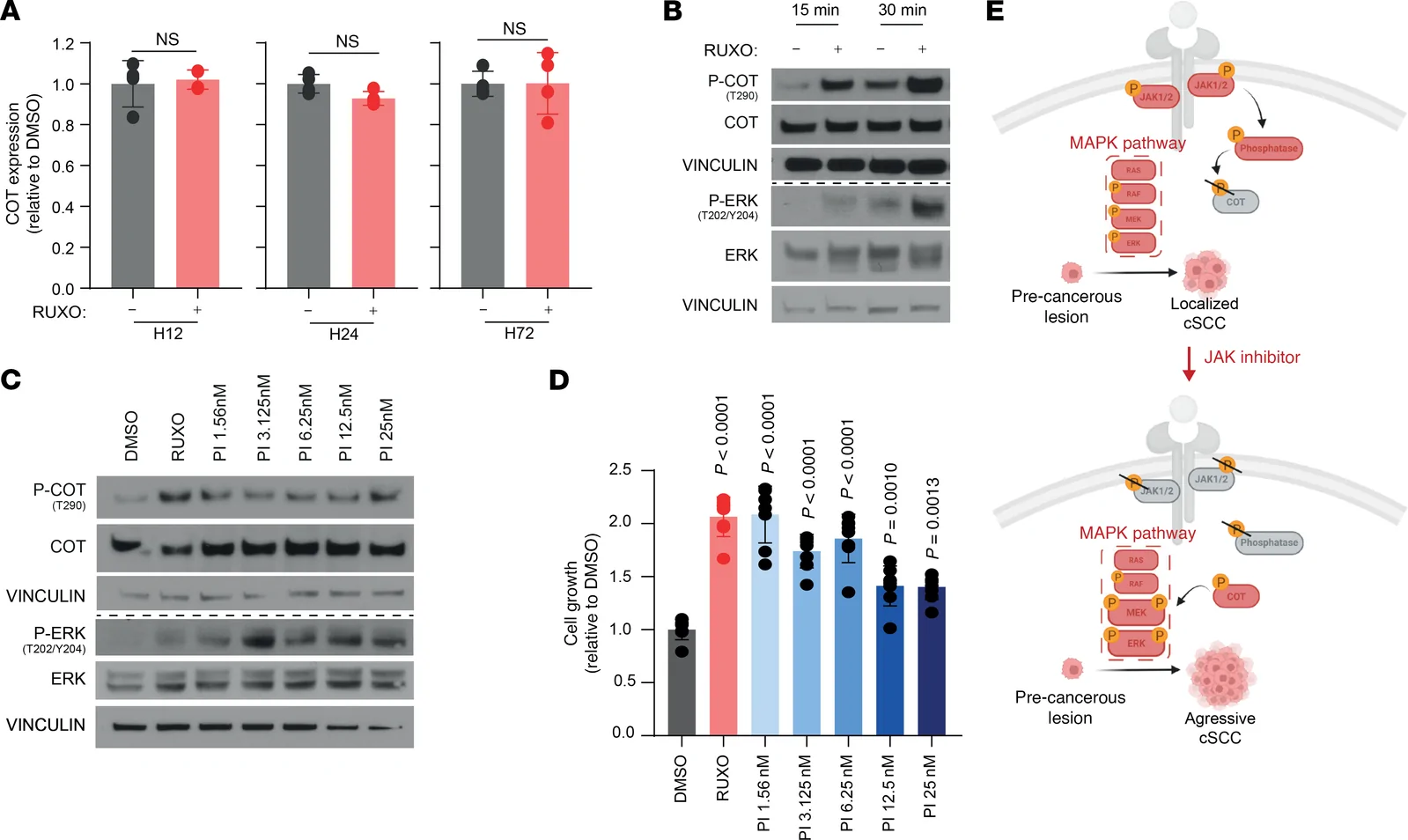
Ruxolitinib-induced COT kinase activation involves a posttranslational regulation mechanism. (**A**) RT-qPCR for COT expression level in A431 cSCC cell line treated with ruxolitinib (3.125 μM) compared with control at the indicated time points. Statistical significance was determined using Mann-Whitney test. Data represent mean ± SD of 4 replicates. (**B**) Phosphorylation level of COT and ERK by Western blot in A431 cSCC cell line treated with ruxolitinib (3.125 μM) compared with DMSO at the indicated time points. (**C**) Phosphorylation level of COT and ERK by Western blot in A431 cSCC cell line treated with ruxolitinib (3.125 μM) or increased doses of serine/threonine phosphatase inhibitor (PI) compared with DMSO. (**D**) Cell growth of A431 cSCC cell line after 6 days of ruxolitinib treatment (3.125 μM) and increased doses of serine/threonine PI compared with DMSO. Statistical significance was determined using 1-way ANOVA. Data represent mean ± SD of 8 replicates. This experiment was performed 2 times. (**E**) Schematic of the proposed model for MAPK pathway activation mechanism under ruxolitinib treatment. Activated proteins are in red, and inactive ones are represented in gray. Created in BioRender.

**Figure 9:**
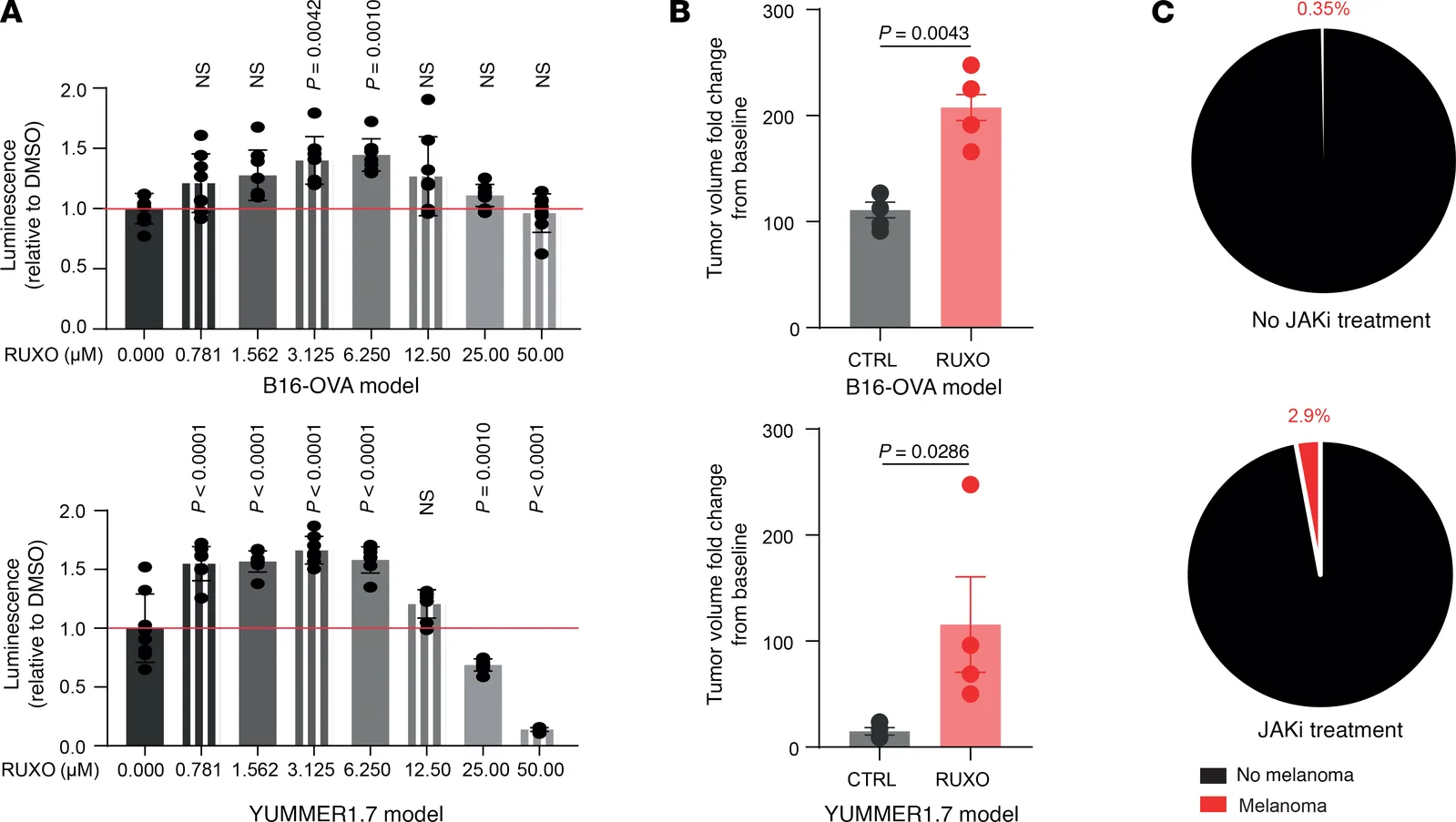
Ruxolitinib-mediated oncogenic effects result in an increased risk of melanoma. (**A**) Cell growth of B16-OVA and YUMMER1.7 melanoma cell lines treated with increasing doses of ruxolitinib for 3 days. Statistical significance was determined using 1-way ANOVA. Data represent mean ± SD of 8 replicates. This experiment was performed 2 times. Conditions above the red horizontal line indicate increased proliferation under ruxolitinib compared with DMSO. (**B**) Tumor volume change from baseline in 2 melanoma mouse models (B16-OVA and YUMMER1.7) treated with ruxolitinib (90 mg/kg bid; *n* = 6 for B16-OVA and *n* = 4 for YUMMER1.7) compared with vehicle-treated control mice (*n* = 5 for B16-OVA and *n* = 4 for YUMMER1.7) after 10 days of treatment. Statistical significance was determined using Mann-Whitney test. Data represent mean ± SEM. (**C**) Proportion of melanoma (in red) among MPN patients treated with JAK inhibitor or not.

## Version 1 11/02/2026

Electronic publication
