# Supplementary material for: JAK1/2 inhibition promotes aggressive cutaneous carcinogenesis through COT kinase–mediated alternative MAPK pathway activation
Source: J Clin Invest. 2026 Nov 2;136(21):e199141. doi: 10.1172/JCI199141 (PMC13722398; doi:10.1172/JCI199141)
Supplement: Supplementary file 1 [file jci-136-199141-s182.pdf]

Figure S1

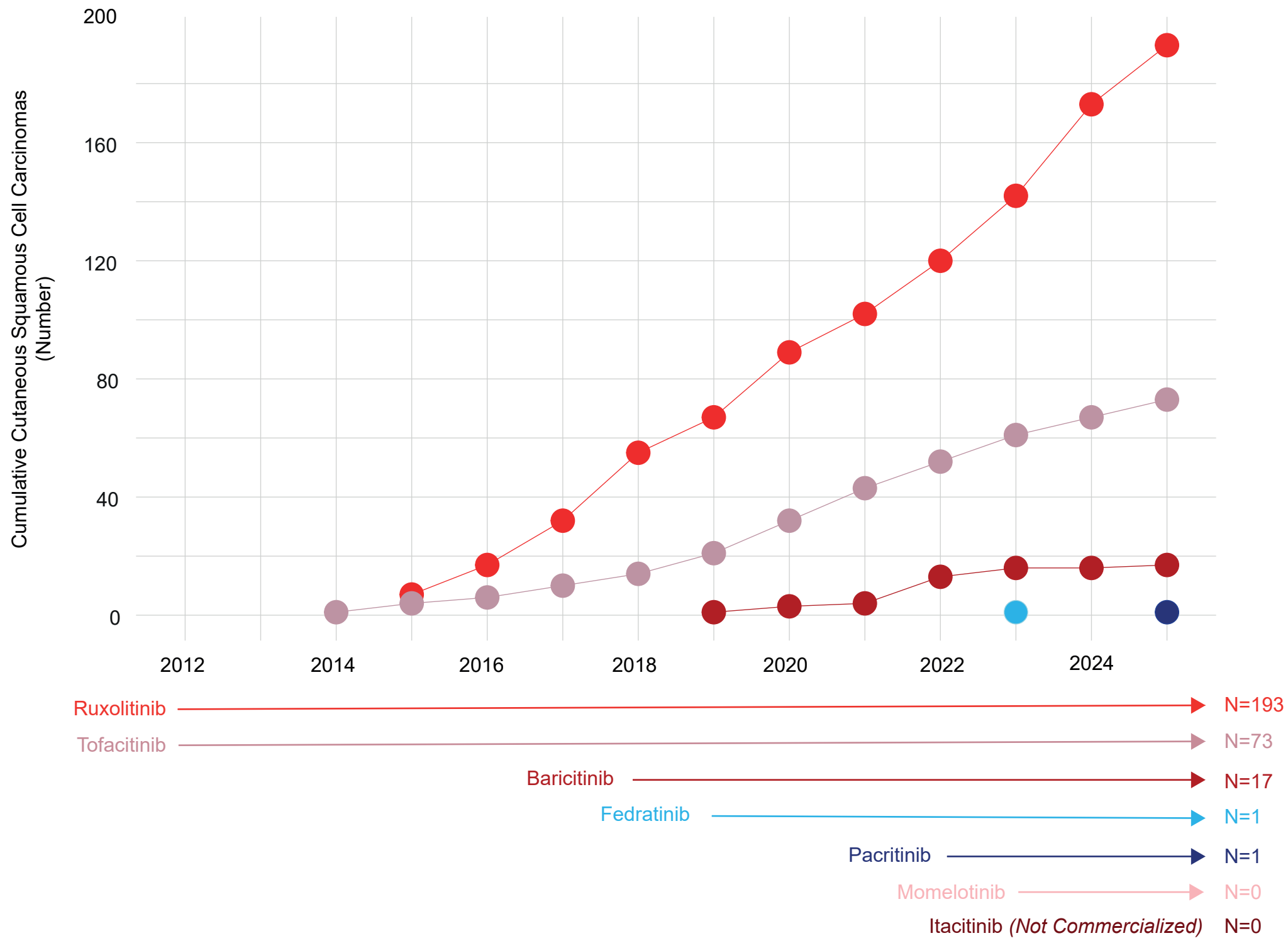

Cumulative Cutaneous Squamous Cell Carcinomas (Number)
200
160
120
80
40
0
2012
2014
2016
2018
2020
2022
2024
Ruxolitinib
N=193
Tofacitinib
N=73
Baricitinib
N=17
Fedratinib
N=1
Pacritinib
N=1
Momelotinib
N=0
Itacitinib (Not Commercialized)
N=0

Figure S2

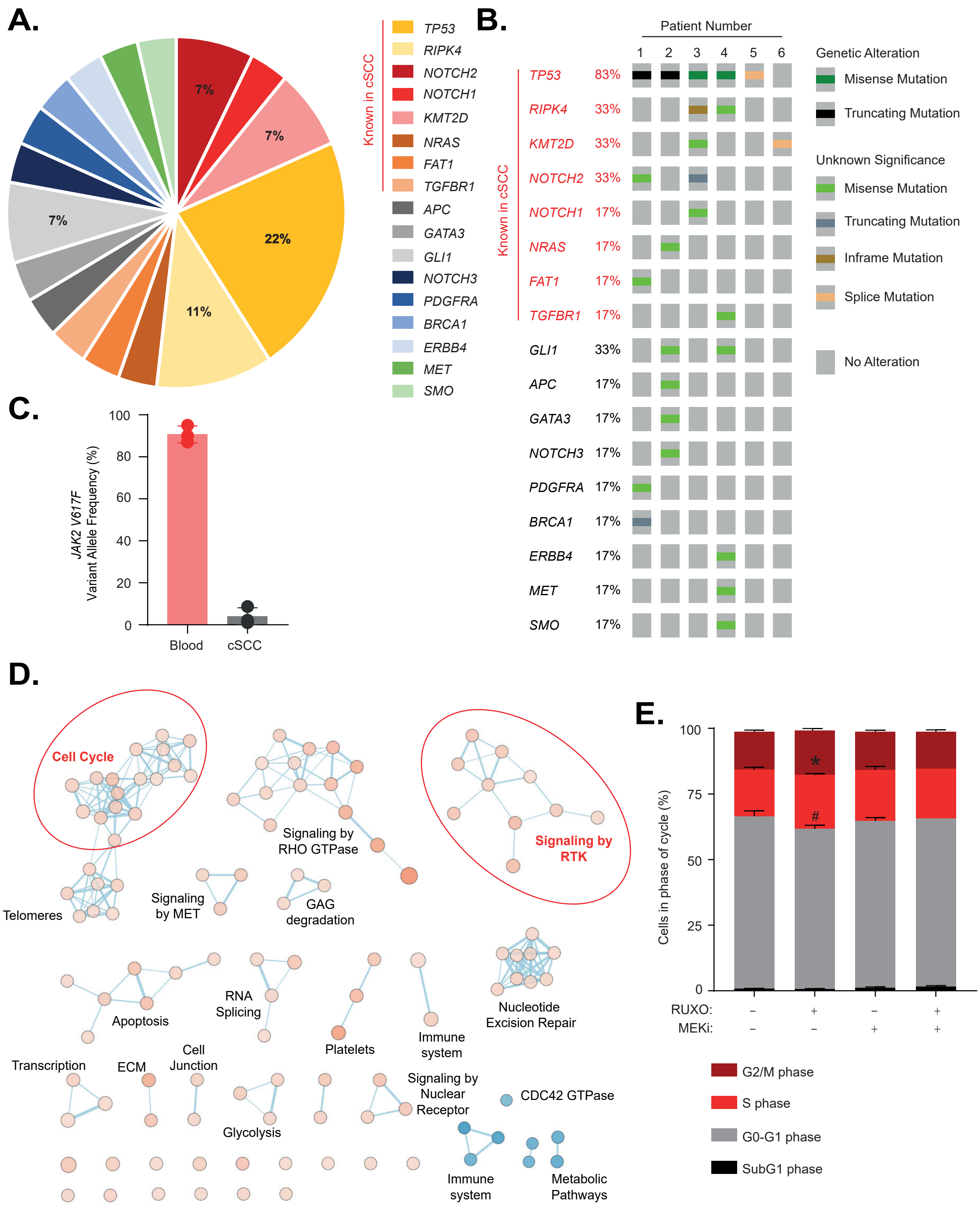
A.
7%
7%
22%
11%
7%
Known in cSCC
TP53
RIPK4
NOTCH2
NOTCH1
KMT2D
NRAS
FAT1
TGFBR1
APC
GATA3
GLI1
NOTCH3
PDGFRA
BRCA1
ERBB4
MET
SMO
B.
Patient Number
1 2 3 4 5 6
TP53 83%
RIPK4 33%
KMT2D 33%
NOTCH2 33%
NOTCH1 17%
NRAS 17%
FAT1 17%
TGFBR1 17%
GLI1 33%
APC 17%
GATA3 17%
NOTCH3 17%
PDGFRA 17%
BRCA1 17%
ERBB4 17%
MET 17%
SMO 17%
Known in cSCC
Genetic Alteration
Misense Mutation
Truncating Mutation
Unknown Significance
Misense Mutation
Truncating Mutation
Inframe Mutation
Splice Mutation
No Alteration
C.
JAK2 V617F Variant Allele Frequency (%)
100
80
60
40
20
0
Blood
cSCC
D.
Cell Cycle
Signaling by RHO GTPase
Signaling by RTK
Telomeres
Signaling by MET
GAG degradation
Apoptosis
RNA Splicing
Nucleotide Excision Repair
Platelets
Immune system
Transcription
ECM
Cell Junction
Glycolysis
Signaling by Nuclear Receptor
CDC42 GTPase
Immune system
Metabolic Pathways
E.
Cells in phase of cycle (%)
100
75
50
25
0
*
#
RUXO: – + – +
MEKi: – – + +
G2/M phase
S phase
G0-G1 phase
SubG1 phase

Figure S3

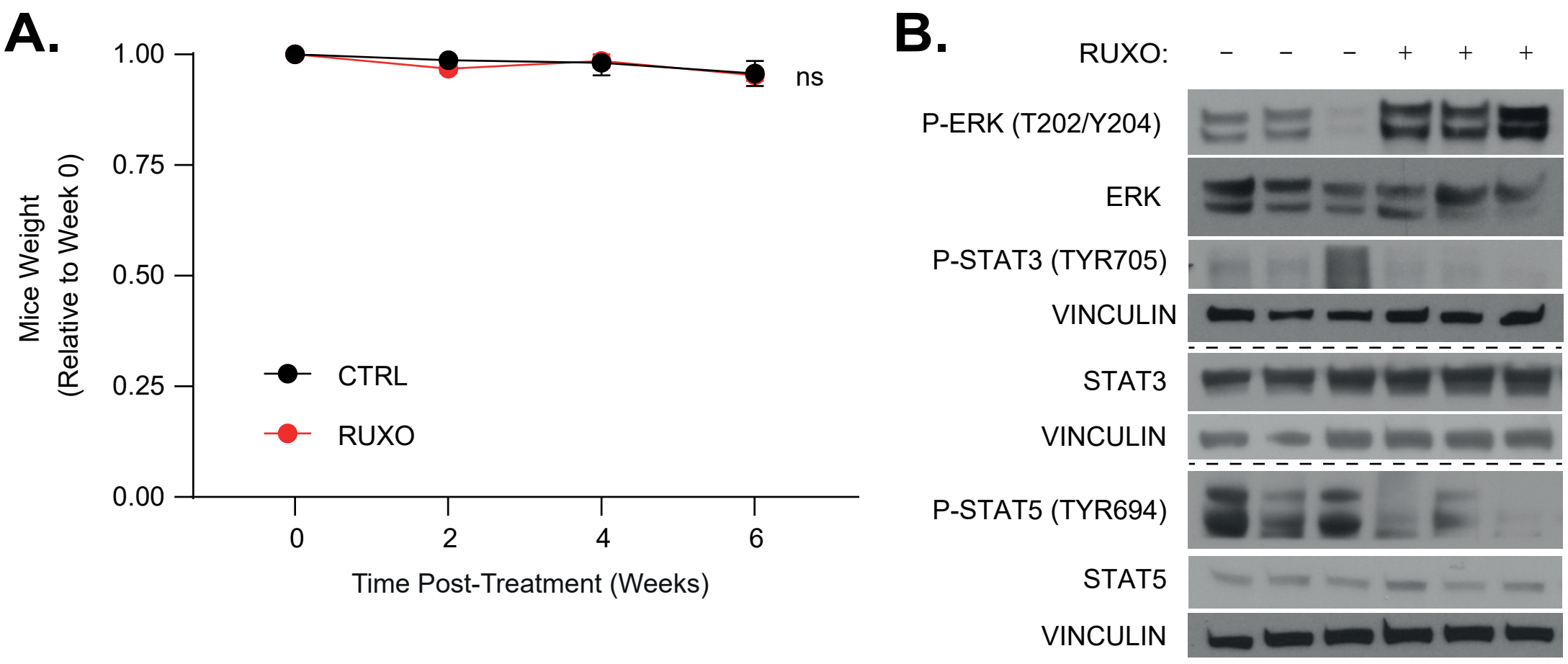

A.
1.00
0.75
0.50
0.25
0.00
Mice Weight
(Relative to Week 0)
ns
CTRL
RUXO
0
2
4
6
Time Post-Treatment (Weeks)
B.
RUXO:
–
–
–
+
+
+
P-ERK (T202/Y204)
ERK
P-STAT3 (TYR705)
VINCULIN
STAT3
VINCULIN
P-STAT5 (TYR694)
STAT5
VINCULIN

SUPPLEMENTARY LEGEND FIGURE

Figure S1. Cutaneous squamous cell carcinomas reported under different JAK inhibitors over time (between 2014 and 2025) according to the international pharmacovigilance database (Vigibase). Each arrow represents the duration of each JAK inhibitor use since its approval. JAK1/2 inhibitors, JAK2 specific inhibitors and JAK1 specific inhibitor are represented in red, blue and brown respectively. The total number of cutaneous squamous cell carcinomas reported for each JAK inhibitor is reported next to each arrow.

Figure S2. Patient tumor molecular profiles and mouse tumor proteomics of ruxolitinib mediated cutaneous squamous cell carcinoma (cSCC).

(A, B) Pie chart **(A)** and OncoPrint plot **(B)** depicting the molecular landscape of ruxolitinib mediated aggressive cSCC in MPN patients (n=6), using a targeted next generation sequencing panel (n=40 genes). Genes highlighted in red are known to be mutated in cSCC. Pie chart describes mutation percentages among all patients while OncoPrint describes mutation percentages per patient. **(C)** *JAK2 V617F* mutation Variant Allele Frequency (VAF) in blood and in ruxolitinib associated cSCC of three MPN patients. **(D)** Network of differentially expressed proteins in cutaneous carcinomas developed by ruxolitinib treated mice (n=5) in comparison to control mice (n=4). Proteins are considered as enriched if log2 fold change > 1 and p-value < 0.05. **(E)** Propidium iodide cell cycle analysis of A431 cSCC cell line after three days of DMSO, ruxolitinib (3.125µM), CI-1040 (3µM) or CI-1040 and ruxolitinib treatment. Statistical significance determined using Mann-Whitney test. Data represent mean ± SD of four replicates. (*) in comparison to DMSO condition for G2/M phase. (#) in comparison to DMSO condition for S phase. * p<0.05, # p<0.05.

Figure S3. Tolerance and pharmacodynamics of ruxolitinib treatment in FvB/N mice

(A) Weight percentage in FvB/N mice treated with ruxolitinib (n=7) in comparison to vehicle treated control mice (n=6) during 6 weeks. Statistical significance determined using Mann-Whitney test in comparison to control conditions. Error bars represent mean of six to seven mice per group ± SEM.

ns=Not Significant. **(B)** Phosphorylation level of ERK, STAT3 and STAT5 by western blot in ruxolitinib associated mice tumors (n=3) in comparison to control mice tumors (n=3).

SUPPLEMENTARY TABLES

Table S1. Characteristics of cutaneous squamous cell carcinoma (cSCC) cases potentially related to ruxolitinib as identified in Vigibase.

Characteristics	PT, n=76 (%)
**Reporting year**	
2015	9 (11.8)
2016	14 (18.4)
2017	18 (23.7)
2018	16 (21.1)
2019	9 (11.8)
2020	10 (13.2)
**Age (years)**	
Median [IQR]	73 [66; 77]
<18	0 (0.0)
[18-45[	0 (0.0)
[45-65[	5 (6.6)
[65-75[	18 (23.7)
≥75	10 (13.2)
Missing data	43 (56.6)
**Sex**	
Male	34 (44.7)
Female	10 (13.2)
Missing data	32 (42.1)
**Serious**	75 (98.7)
Death	6 (8.0)
Life threatening	0 (0.0)
Hospitalization or hospitalization prolongation	16 (21.3)
Persistent or significant disability/incapacity	1 (1.3)
Other medically important condition	52 (69.3)
**Time-to-onset (months)**	
Median [IQR]	34.3 [23.3;49.7]
< 6 months	1 (1.3)
[6 - 12[months	3 (4.0)
[12 - 18[months	2 (2.6)
[18 - 24[months	2 (2.6)
[24 - 36[months	4 (5.3)
≥ 36 months	9 (11.8)
Missing data	55 (72.4)

**Associated suspected drugs**	
Hydroxycarbamide	8 (10.5)
Warfarin	0 (0.0)
Amoxicillin	0 (0.0)
Atenolol	2 (2.6)
**Indications[a]**	
Myelofibrosis	41 (53.9)
Polycythemia Vera	33 (43.4)
Myeloproliferative neoplasm	1 (1.3)
Essential thrombocythemia	2 (2.6)
Missing data	2 (2.6)

[a] ICSRs can report more than one indication, mainly myelofibrosis and polycythemia vera.

ICSRs: individual case safety reports; IQR: interquartile range; PT: Preferred Terms.

Table S2. Signals of disproportionate reporting for cutaneous squamous cell carcinoma (cSCC) and ruxolitinib exposure in comparison with other treatments.

	n	$n_{reaction}$	n_{drug}	PRR	95%CI*	IC	95%CI**
**Comparator: antineoplastic and immunomodulating agents**							
**At least one PT**	**76**	**2067**	**34234**	**4,88**	**[3.88; 6.14]**	**2,21**	**[1.83; 2.48]**
Metastatic	3	18	34234	25,56	[7.4; 88.29]	2,45	[0.38; 3.66]
Non metastatic	73	2051	34234	4,72	[3.73; 5.96]	2,16	[1.77; 2.44]
**Comparator: drugs with the same indication**							
**At least one PT**	**76**	**138**	**34234**	**7**	**[5.01; 9.80]**	**1,86**	**[1.48; 2.14]**
Metastatic	3	3	34234	-	-	1,89	[-0.18; 3.09]
Non metastatic	73	135	34234	6,73	[4.80; 9.44]	1,83	[1.45; 2.11]
**Comparator: hydroxycarbamide**							
**At least one PT**	**76**	**136**	**34234**	**4,72**	**[3.36; 6.62]**	**1,38**	**[1.00; 1.66]**
Metastatic	3	3	34234	-	-	1,62	[-0.45; 2.83]
Non metastatic	73	133	34234	4,53	[3.22; 6.37]	1,36	[0.97; 1.64]

PRR: proportional reporting ratio; IC: information component; 95%CI*: 95% confidence interval; 95%CI**: 95% credibility interval

PT: Preferred Terms.

Table S3. Characteristics of patients diagnosed with myeloproliferative neoplasms.

	All patients (n=1004)	JAKi treated patients (n=136)	NON-JAKi treated patients (n=868)
**Age (years), median (IQR)**	51 [40; 64]	58 [45; 66]	50 [39; 63]
**Female, nb (%)**			
Yes	546 (54.38%)	61 (44.85%)	485 (55.88%)
No	458 (45.62%)	75 (55.15%)	383 (44.12%)
**MPN initial subtype, nb (%)**			
PMF	133 (13.25%)	50 (36.76%)	83 (9.56%)

ET	453 (45.12%)	35 (25.74%)	418 (48.16%)
PV	337 (33.57%)	46 (33.82%)	291 (33.53%)
MDS/MPN	11 (1.10%)	4 (2.94%)	7 (0.81%)
MPN unclassified	70 (6.97%)	1 (0.74%)	69 (7.95%)

MPN: Myeloproliferative neoplasms; PV: Polycythemia Vera; ET: Essential Thrombocythemia; PMF: Primary myelofibrosis; MDS: Myelodysplastic Syndrome; JAKi: JAK inhibitor; nb: number.

Table S4. Univariate and multivariate analysis evaluating cutaneous squamous cell carcinoma (cSCC) associated factors.

Variables		Univariate analysis			Multivariate analysis	
	HR	95% CI	pval	HR	95% CI	pval
**Age**	1.05	[1.03; 1.06]	**<0.001**	1.04	[1.02; 1.06]	**0.001**
**Female**						
Yes	1	.	.	1	.	.
No	4.65	[1.90; 11.36]	**0,001**	3.52	[1.39; 8.93]	**0.008**
**MPN initial subtype**						
PMF	1	.	.			
ET	0.28	[0.10; 0.74]	**0.01**			
PV	0.52	[0.21; 1.28]	0.154			
MDS/MPN	9.6E-09	[2.99E-09; 3.08E-08]	**<0.001**			
MPN unclassified	9.47E-09	[3.83E-09; 2.34E-08]	**<0.001**			
**JAKi treatment**						
No	1	.	**.**	1	.	.
Yes	10,56	[4.70; 23.72]	**<0.001**	6.52	[2.75; 15.46]	**<0.001**

MPN: Myeloproliferative neoplasms; PV: Polycythemia Vera; ET: Essential Thrombocythemia; PMF: Primary myelofibrosis; MDS: Myelodysplastic Syndrome; JAKi: JAK inhibitor; HR: Hazard ratio; CI: Confidence interval; pval: p-value.

Table S5. Characteristics of patients diagnosed with aggressive cutaneous squamous cell carcinoma (cSCC) under JAK inhibitor treatment.

	**Patients (n=6)**
**DERMATOLOGICAL CHARACTERISTICS**	
**Age at cSCC diagnosis (years), median (IQR)**	71.5 [68;85]
**History of actinic keratosis, nb (%)**	
No	1 (16.6%)
Yes	5 (83.4%)
**cSCC number, median (IQR)**	1.5 [1;11]
**T3 or T4 tumor, nb (%)**	
No	3 (50%)
Yes	3 (50%)
**Lymph node infiltration, nb (%)**	

No	3 (50%)
Yes	3 (50%)
**Visceral metastasis, nb (%)**	
No	5 (83.4%)
Yes	1 (16.6%)
**Ear or lip location, nb (%)**	
No	2 (33.3%)
Yes	4 (66.7%)
**Clinical size > 2cm, nb (%)**	
No	2 (33.3%)
Yes	3 (50%)
Unknown	1 (16.7%)
**Poor differenciation degree, nb (%)**	
No	2 (33.3%)
Yes	4 (66.7%)
**Vertical thickness extension > 2mm, nb (%)**	
No	0 (0%)
Yes	2 (33.3%)
Unknown	4 (66.7%)
**Extension beyond hypodermis, nb (%)**	
No	4 (66.7%)
Yes	2 (33.3%)
**Bone extension, nb (%)**	
No	5 (83.4%)
Yes	1 (16.6%)
**Vascular embolism, nb (%)**	
No	3 (50%)
Yes	3 (50%)
**Perineural invasion, nb (%)**	
No	3 (50%)
Yes	3 (50%)
**HEMATOLOGICAL CHARACTERISTICS**	
**Age at MPN diagnosis (years), median (IQR)**	60 [42;73]
**MPN initial subtype, nb (%)**	
PV	3 (50%)
ET	2 (33.3%)
MF	1 (16.6%)
**Driver mutation, nb (%)**	
JAK2 V617F	4 (66.7%)
CALR	1 (16.6%)
MPL	0 (0%)
Triple negative	1 (16.6%)
**Additionnal mutations, nb (%)**	
TET2	2 (33.3%)
ASXL1	1 (16.6%)
KRAS	1 (16.6%)
CBL	1 (16.6%)
SF3B1	1 (16.6%)

SH2B3	2 (33.3%)
TP53	2 (33.3%)
**JAK inhibitor treatment, nb (%)**	
Ruxolitinib	4 (66.7%)
Momelotinib	1 (16.6%)
Pacritinib	1 (16.6%)
**Thrombotic and/or hemorraghic events, nb (%)**	
No	5 (83.4%)
Yes	1 (16.6%)
**MF transformation, nb (%)**	
No	1 (16.6%)
Yes	5 (83.4%)
**AML/MDS transformation, nb (%)**	
No	5 (83.4%)
Yes	1 (16.6%)

cSCC: cutaneous squamous cell carcinoma; MPN: Myeloproliferative Neoplasms; ET: Essential Thrombocythemia; PV: Polycythemia Vera; MF: Myelofibrosis; MDS: Myelodysplastic Syndrome; IQR: Interquartile range; nb: number.

Table S6. Histological characteristics of murine cutaneous squamous cell carcinoma (cSCC).

	**Control n=5**	**Ruxolitinib n=11**
**Tumor differentiation, n (%)**		
Well	2 (40%)	3 (27%)
Moderate	1 (20%)	5 (46%)
Poor	2 (40%)	3 (27%)
**Tumor cytology, n (%)**		
Fusiform	2 (40%)	2 (18%)
Epithelioid	3 (60%)	8 (73%)
Epithelioid and fusiform	0	1 (9%)
**Last structure infiltrated in depth, n (%)**		
Dermis	0	1 (9%)
Hypodermis	1 (20%)	1 (9%)
Muscle	2 (40%)	3 (27%)
Beyond muscle	2 (40%)	6 (55%)
**Tumor necrosis, n (%)**		
Yes	0 (0%)	2 (18%)
No	5 (100%)	9 (82%)
**Vacular embolism, n (%)**		
Yes	0 (0%)	1 (9%)
No	5 (100%)	10 (91%)
**Perineural sheathing, n (%)**		
Yes	0 (0%)	0 (0%)
No	5 (100%)	11 (100%)

Table S7. Cutaneous squamous cell carcinomas reported under different JAK inhibitors between 2014 and 2025 according to the international pharmacovigilance database (Vigibase). *JAKi: JAK inhibitor*

Year	2014	2015	2016	2017	2018	2019	2020	2021	2022	2023	2024	2025	Total of Cases per JAKi
**JAK1/2 Inhibitor**													
RUXOLITINIB		7	10	15	23	12	22	13	18	22	31	20	193
TOFACITINIB	1	3	2	4	4	7	11	11	9	9	6	6	73
BARICITINIB						1	2	1	9	3	0	1	17
MOMELOTINIB													0
**JAK2 Inhibitor**													
FEDRATINIB										1			1
PACRITINIB												1	1
**JAK1 Inhibitor**													
ITACITINIB													0
**Total of Cases per Year**	1	10	12	19	27	20	35	25	36	35	37	28	285

Table S8. List of differentially abundant proteins in ruxolitinib mice tumors (n=5) compared to control mice tumors (n=4). Data is too large to fit in a word and is shown as an excel file.

Table S9. List of differentially abundant phosphosites in ruxolitinib mice tumors (n=5) compared to control mice tumors (n=4). Data is too large to fit in a word and is shown as an excel file.

Table S10. List of differentially abundant phosphosites within the MAPK pathway in ruxolitinib mice tumors (n=5) compared to control mice tumors (n=4).

Phosphos-protein	p-value	Fold-Change
Map2k2-Phospho:S226	1.60E-06	38.5763
Map2k2-Phospho:S222	0.000260413	25.739
Mapk14-Phospho:Y182	0.00654207	4.43028
Map4k4-Phospho:S629	0.00837377	2.48745
Map3k2-Phospho:T337	0.0087136	3.18123
Map4k4-Phospho:S795	0.0166956	2.98559

Map3k2-Phospho:T339	0.0167824	3.1553
Map3k2-Phospho:S331	0.0241279	3.22336
Map3k2-Phospho:S297	0.0354547	2.71297
Map4k4-Phospho:S794	0.0409649	2.29663
Mapk3-Phospho:Y205	0.0417339	5.30324

Table S11. List of the 40 genes included in the Next Generation Sequencing (NGS) panel for cSCC samples molecular landscape.

Cutaneous squamous cell carcinoma NGS panel (n=40 genes)
AJUBA (JUB)
BBS9
BRAF
CARD11
CASP8
CCND1
CDKN2A
COBLL1
DCLK1
DCLRE1A
EGFR
EIF2D
FAT1
FBXO21
HRAS
KMT2C (MLL3)
KMT2D (MLL2)
MMP2
NFE2L2
NOTCH1
NOTCH2
NOTCH4
NRAS
OPN3
PARD3
PEG10
PIK3CA
RAC1
RASA1
RBM46
RIPK4
SEC31A

SMAD4
SMAD9
SNX25
STK19
TGFBR1
TP53
VEGF
ZNF644

Table S12. List of shRNA constructs targeting *COT (MAP3K8), JAK1 and JAK2* – sequences.

Designation	shRNA 97-mer oligo sequence
Sh*COT#1*	TGCTGTTGACAGTGAGCGCAGAGATTGATTTATTAATTAATAGTGAAGCCA CAGATGTATTAATTAATAAATCAATCTCTTTGCCTACTGCCTCGGA
Sh*COT#2*	TGCTGTTGACAGTGAGCGCAAGTATGGAATATTCATTTTATAGTGAAGCCA CAGATGTATAAAATGAATATTCCATACTTATGCCTACTGCCTCGGA
Sh*COT#3*	TGCTGTTGACAGTGAGCGCGAGATTGATTTATTAATTAAATAGTGAAGCCA CAGATGTATTTAATTAATAAATCAATCTCTTGCCTACTGCCTCGGA
Sh*JAK1#1*	TGCTGTTGACAGTGAGCGCCCAGATATTGTTTCAGAAAAATAGTGAAGCC ACAGATGTATTTTTCTGAAACAATATCTGGATGCCTACTGCCTCGGA
Sh*JAK1#2*	TGCTGTTGACAGTGAGCGCCCATGCATTCTTAAAATATTATAGTGAAGCCA CAGATGTATAATATTTTAAGAATGCATGGTTGCCTACTGCCTCGGA
Sh*JAK1#3*	TGCTGTTGACAGTGAGCGAAGAATGTTTAATGCAATCTAATAGTGAAGCC ACAGATGTATTAGATTGCATTAAACATTCTGTGCCTACTGCCTCGGA
Sh*JAK1#4*	TGCTGTTGACAGTGAGCGCTCCAGATATTGTTTCAGAAAATAGTGAAGCC ACAGATGTATTTTCTGAAACAATATCTGGATTGCCTACTGCCTCGGA
Sh*JAK2#1*	TGCTGTTGACAGTGAGCGCCCATAGTTAATCTATAATTAATAGTGAAGCCA CAGATGTATTAATTATAGATTAACTATGGATGCCTACTGCCTCGGA

Sh*JAK2#2*	TGCTGTTGACAGTGAGCGCTAAGATGTTCAGATAATTGAATAGTGAAGCC<br>ACAGATGTATTCAATTATCTGAACATCTTAATGCCTACTGCCTCGGA
Sh*JAK2#3*	TGCTGTTGACAGTGAGCGATGGAACAATAATGTAAATCAATAGTGAAGCC<br>ACAGATGTATTGATTTACATTATTGTTCCAGTGCCTACTGCCTCGGA
Sh*JAK2#4*	TGCTGTTGACAGTGAGCGCAGATACATATCTGAAAAAGAATAGTGAAGCC<br>ACAGATGTATTCTTTTTCAGATATGTATCTATGCCTACTGCCTCGGA

Table S13. List of antibodies used for Western-blot.

Target	Conjugate	Reference	Manufacturer
Phospho-ERK1/2 (Thr202/Tyr204)	No	9101	Cell signaling
ERK1/2	No	9102	Cell signaling
Phospho-COT (Thr290)	No	PA5-104891	Thermofisher
COT	No	PA5-95371	Thermofisher
Phospho-RAF (Ser338)	No	9427	Cell signaling
RAF	No	53745	Cell signaling
Phospho-STAT3 (Tyr705)	No	9138	Cell signaling
STAT3	No	4904T	Cell signaling
Phospho-STAT5 (Tyr694)	No	9351	Cell signaling

STAT5	No	94205	Cell signaling
JAK1	No	3344	Cell signaling
JAK2	No	3230	Cell signaling
VINCULIN	No	ab219649	Abcam
Anti-rabbit HRP	HRP	7074	Cell signaling

SUPPLEMENTARY MATERIAL AND METHOD

MURINE SAMPLES HISTOLOGICAL ANALYSIS

Each sample was fixed in Formol 4% (Sigma) during 24h, dehydrated in ethanol 70%, paraffin embedded, and sliced using a microtome prior to HE (Hematoxylin and Eosin) staining. This procedure was performed by HISTIM platform (Cochin institute, Paris, FRANCE). Samples were then analyzed by a blinded specialized dermato-pathologist with expertise in cSCC. cSCC histological diagnosis, histological subtype, tumor differentiation, infiltration depth, presence of vascular embolism, necrosis and peri-neural sheathing were evaluated. Representative pictures were obtained using a nanozoomer 2.0 (Hamamatsu).

IMMUNOFLUORESCENCE

***Immunostaining* –** Tumors were directly embedded in OCT (#2545932, UGAP) and cut with a cryotome (10µm slides). Tissue samples were fixed in 4% paraformaldehyde (16% PFA diluted with PBS, Electron Microscopy Sciences (#D15710)) during 15 min, washed 3 times with PBS and permeabilized with 0.3% Triton (Sigma-Aldrich). After permeabilization, slides were washed 3 times with PBS and blocked with a solution of 5% BSA (Bovine Serum Albumin, Sigma-Aldrich #1003498240) during 30 min. Slides were next stained with primary antibody overnight at 4°C using a polyclonal rabbit anti-phospho-Erk1/2 (Thr202/Tyr204) antibody (#9101, Cell Signaling), diluted at 1:200. After 3 washes with PBS, Alexa Fluor TM secondary antibody 647nm-conjugated goat anti-rabbit (#4414 Cell Signaling) was applied at 1:1000 dilution. Coverslips were mounted using a

ProLong™ Gold DAPI mounting medium (#P36931, Thermofisher) for both nuclear staining and mounting.

Immunofluorescence imaging and quantification- Fixed tissue images were processed using a confocal Zeiss LSM 800 Airy Scan microscope (Saint Louis Research Institute platform) equipped with a dedicated Zeiss Zen desk interface. Image acquisition was performed using a 63X 1.40-NA oil objective. Three randomly and representative selected positions were captured for each tumor. Binary masks were generated using the Fiji platform to measure the intensity of pERK signaling. Signal intensity measurement was quantified by calculating the mean of three distinct measurements.

PHOSPHOPROTEOMIC ANALYSIS

***Cell lysis* -** Frozen cSCC mice tumors were digested mechanically and lysed with urea lysis buffer (8M of urea, Sigma #U5378, 50mM of ammonium bicarbonate, Sigma #A6141, with protease and phosphatase inhibitor cocktail, Thermofisher #78440).

***Sonication* -** Cell lysate was sonicated to reduce viscosity**.**

***Protein quantification* -** Total protein was quantified with QuantiPro™ BCA Assay Kit (Sigma #QPBCA-1KT).

***Denaturation* -** Disulfide bonds of 200µg total protein were reduced by addition of 5mM of Dithiothreitol (Sigma #D0632) and incubation at 55°C for 1 hour. 10mM of iodoacetamide (GE Healthcare #RPN6302) was added to the sample and incubated at room temperature for 30 min.

***Digestion* -** Protein were digested with 1µg of trypsin (Promega #V5072) for 100µg of total protein at 37°C for 16 hours under constant shaking (180 rpm).

***Desalting* -** Final concentration of 1% trifluoroacetic acid (TFA) (Sigma #299537) was next added for desalting step during 15 min on ice followed by a centrifugation for 1min at 1800 x g, 4°C prior. Supernatant was added into the Sep Pak C18 50mg (Waters #WAT054955) column for further purification. 50% acetate natrium (ACN) (Sigma #14261) with 0.1% of TFA was finally added into the column for peptide elution prior to a lyophilization process.

***Phosphopeptide purification* -** Loading buffer (80% ACN with 5% TFA and 1M of glycolic acid, Sigma #124737) was added to 250 - 300µg lyophilized peptide sample. Each sample sample was treated with 0.25mg TiO 2 (MR-TID002) and 1.25mg ZrO 2 (MR-ZRD002) beads in a 96-well plate and incubated for 30 min at room temperature under constant shaking (600 rpm). Unbound peptides were removed with magnetic separation followed by an additional wash with a loading buffer. Non-specifically bound peptides were washed with wash buffer 1 (80% ACN and 1% TFA) for 2 minutes followed by wash buffer 2 (10% ACN and 0.2% TFA) for 2 minutes under constant shaking (600 rpm).

***Elution and MS* –** Phospho-peptides were eluted from the beads with 1% of ammonium hydroxide (NH 4 OH, Sigma #338818). Eluted products were transferred into LoBind Eppendorf tube containing 20% formic acid HCO 2 H (Sigma #F0507) and ultimately transferred into home-made column tips containing C18 membrane (Waters #WAT054955) prior to mass spectrometry analysis by LC-MS/MS at the Curie institute mass spec facility (Paris, France) using a Thermo Scientific Q Exactive HF-X mass spectrometer with data independent acquisition.

***Proteomic and phosphoproteomic GSEA and KEA analysis* -** MS spectra were processed using spectronaut version 15.6 (Biognosis) and resulting intensity data matrices for proteomic and phosphoproteomic were analyzed using Partek Genomics Suite software (Partek) to generate differential analysis using ANOVA. Gene set enrichment analysis of proteomic data was performed in Cytoscape using the default setting of the Enrichment map application. Kinase enrichment analysis was performed using the kinase enrichment tool provided by phosphositeplus (https://kinase-library.phosphosite.org/ea) with log2 fold change threshold of 1 and p-value threshold of 0.05[26] or the RoKAI online app with default settings[27].

RNA SEQUENCING

***Sample processing* –** Frozen tumors were lysed and homogenized mechanically in RLT buffer. RNA was next extracted using Qiagen RNeasy Mini Kit (#74106) according to the manufacturer's protocol. Sequencing libraries were prepared using TruSeq Stranded mRNA LT Sample Prep Kit (Illumina) and 100-bp paired-end sequenced on a NovaSeq6000 (Illumina) at Macrogen Europe.

The total number of reads for individual samples ranged from 83 million to 97 million with at least 94% of reads > Q30.

Bio-informatic analysis - Quality-control tests for the unmapped reads were performed using the FASTQC software (https://github.com/s-andrews/FastQC). Sequencing reads were pseudo-aligned to the ENSEMBL GRCm38 version 96 of the mouse genome and abundance-quantified using Kallisto (https://pachterlab.github.io/kallisto/). Differential expression analysis was performed using the Sleuth (v0.30.0) R package with a Wald-test between comparisons. The q values were determined using a Benjamini–Hochberg correction of p values. Genes were considered as significantly differentially expressed if qvalue was inferior or egal to 0.05. For GSEA analysis, genes were ranked using their log2 fold change of expression between control and ruxolitinib conditions. GSEA was carried out using MSigDB genesets from C2 common pathways, C5 molecular functions and Hallmark collections. GSEA analysis was performed using the R package fgsea (v1.2.0). The adjusted p value (padj) was determined using a Benjamini–Hochberg correction of p values. Gene sets with padj inferior or egal to 0.25 were considered as significantly enriched pathways. Volcano/barplots and GSEAplot were plotted using R packages ggplot2 and gseaplot2 respectively. Heatmaps were plotted using Phantasus v1.23.12.
