# Supplementary material for: JAK1/2 inhibition promotes aggressive cutaneous carcinogenesis through COT kinase–mediated alternative MAPK pathway activation
Source: J Clin Invest. 2026 Nov 2;136(21):e199141. doi: 10.1172/JCI199141 (PMC13722398; doi:10.1172/JCI199141)
Supplement: Supplementary file 2 [file jci-136-199141-s183.pdf]

Figure 3E

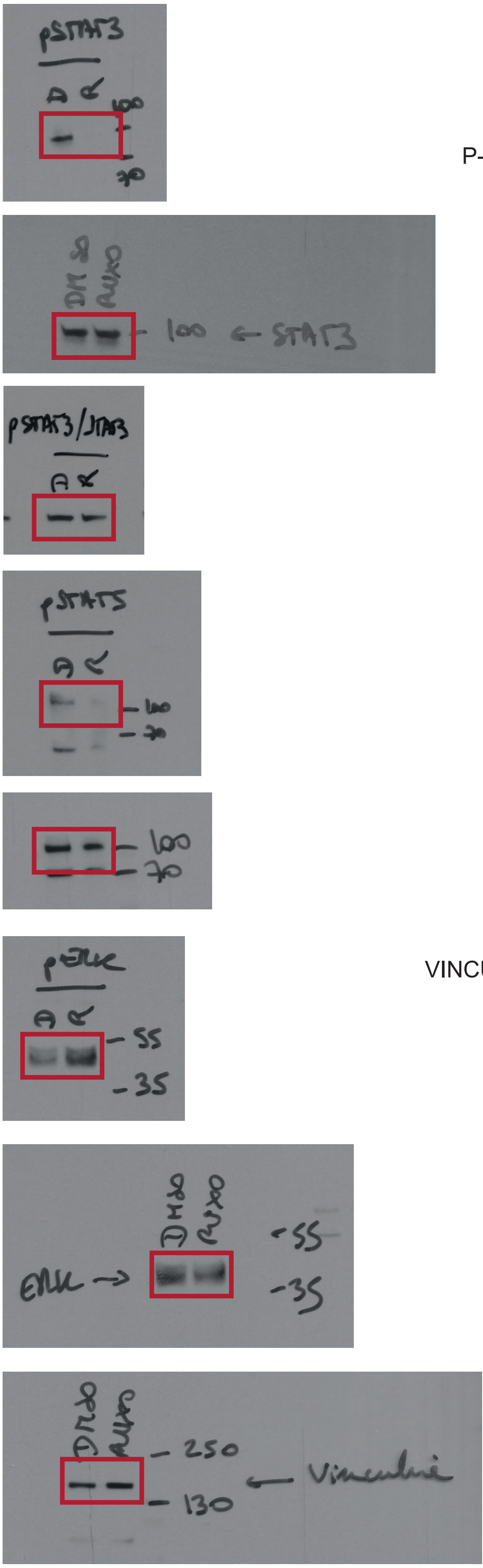

Figure 3G

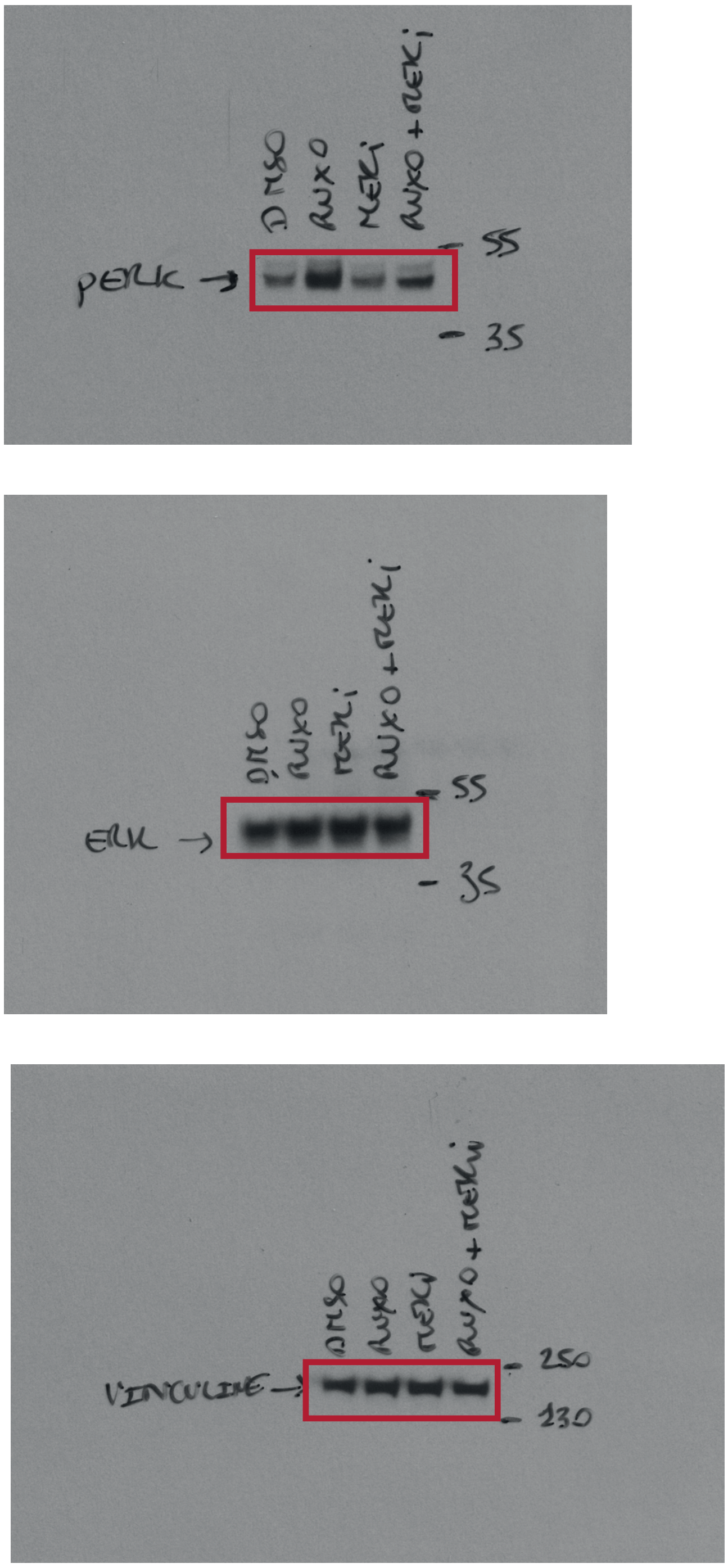

Figure 4B

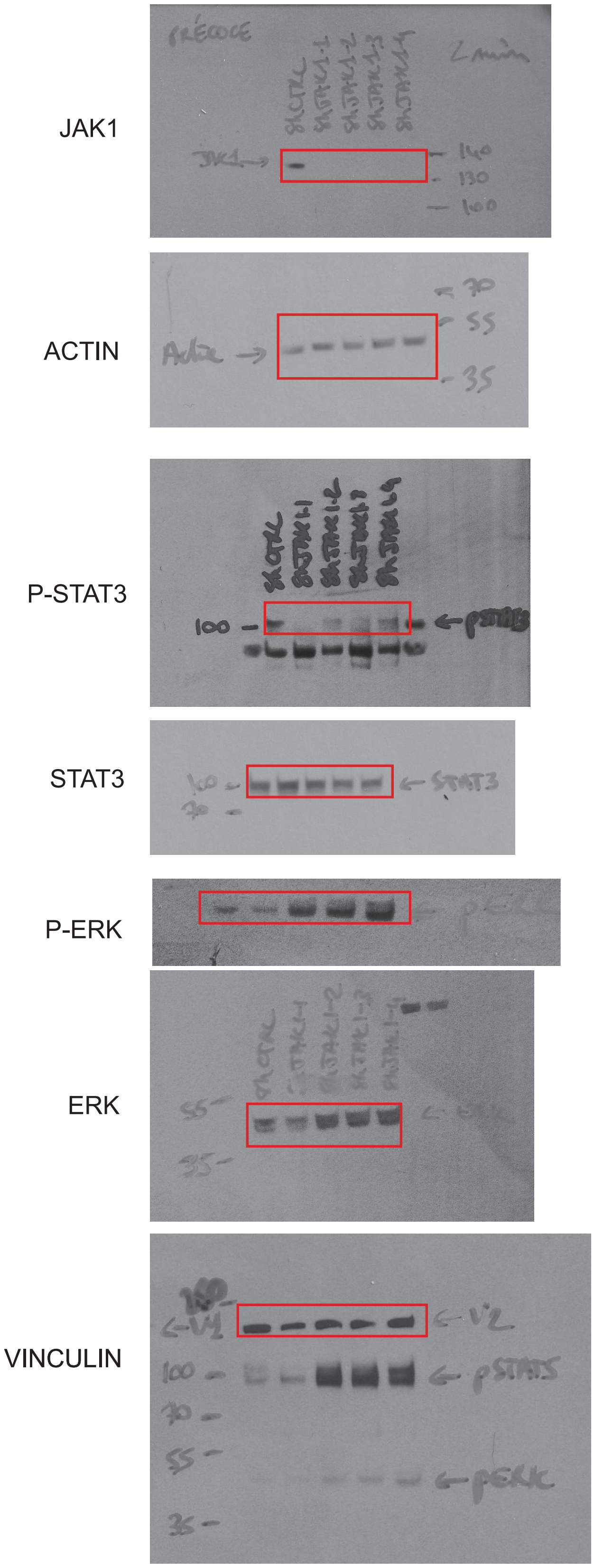

Figure 4E

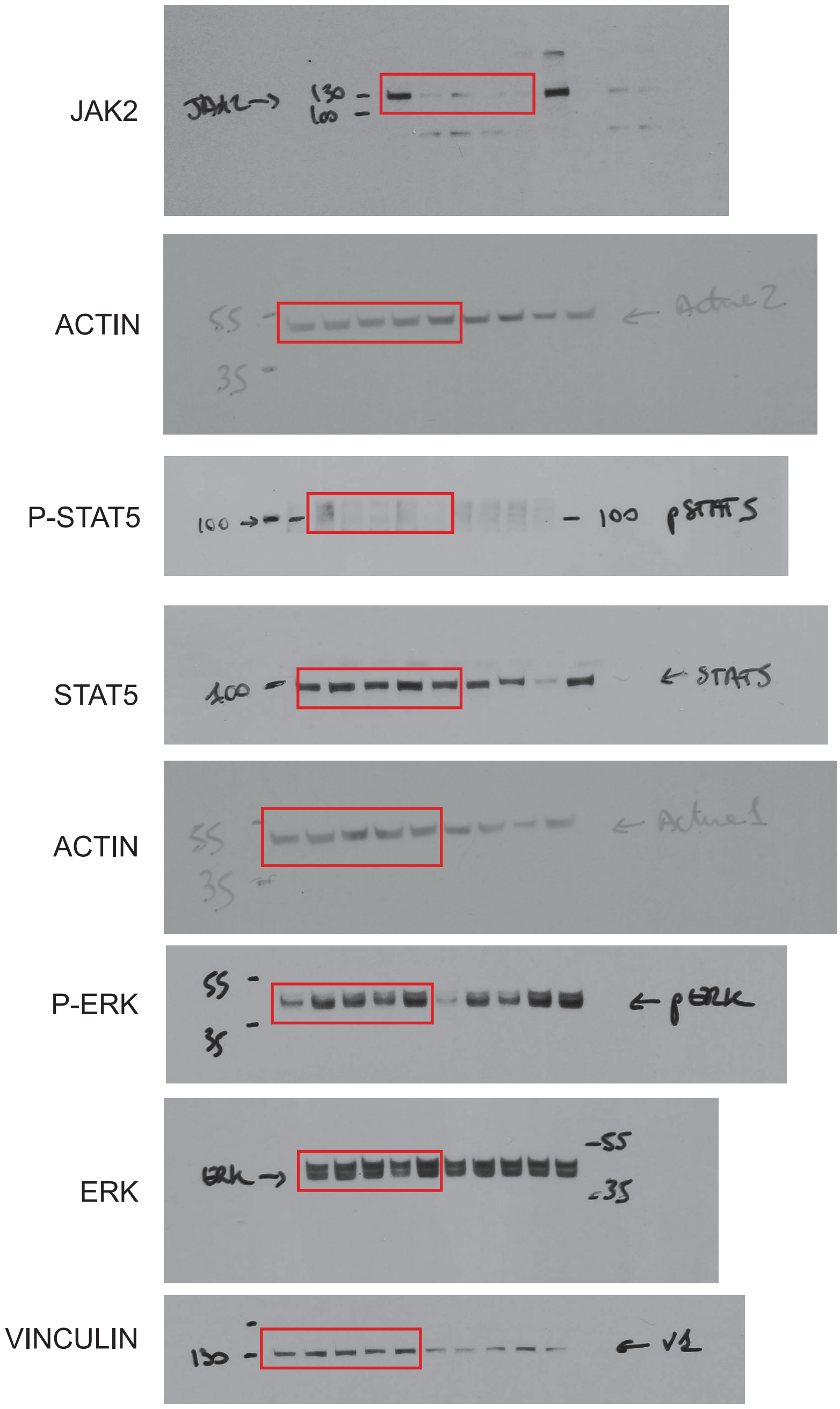

Figure 5B

P-ERK

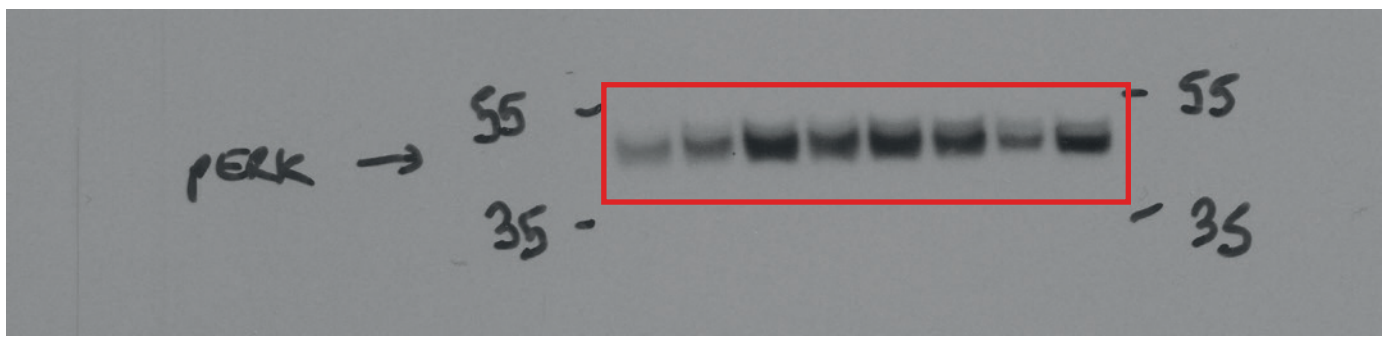

ERK

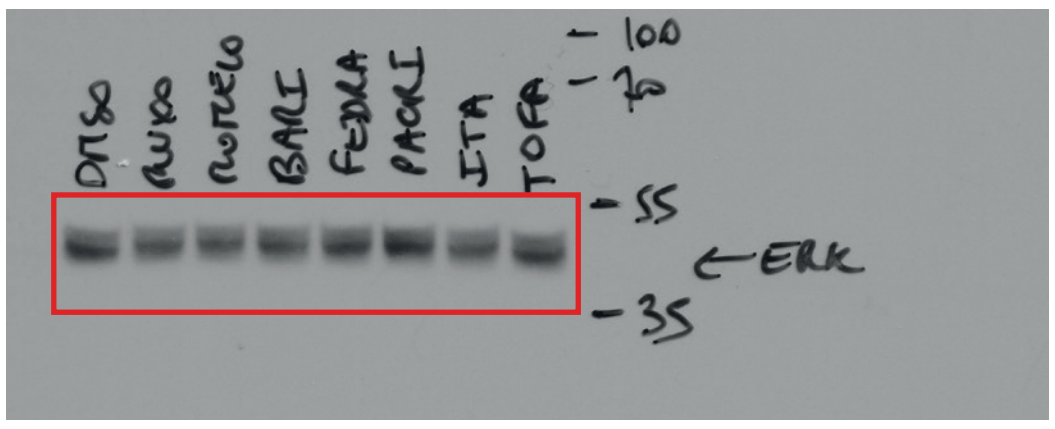

VINCULIN

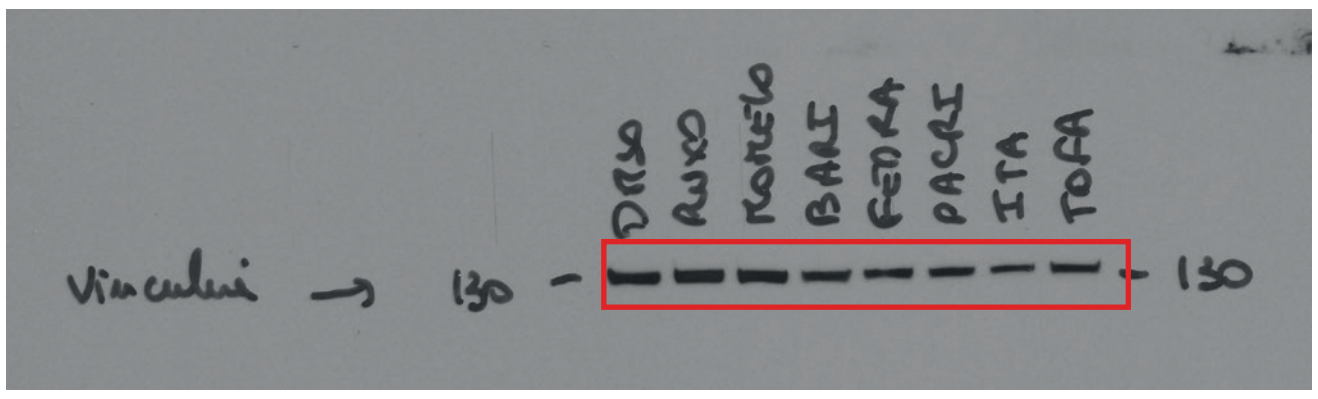

Figure 6D

P-RAF

RAF

P-COT

COT

P-ERK

ERK

VINCULIN

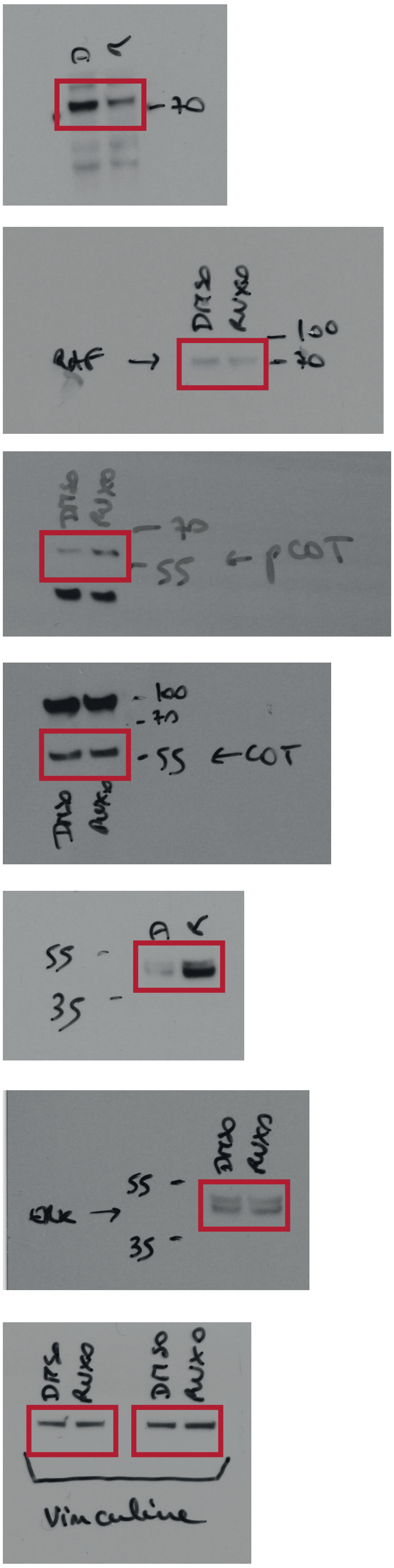

Figure 7E

P-ERK

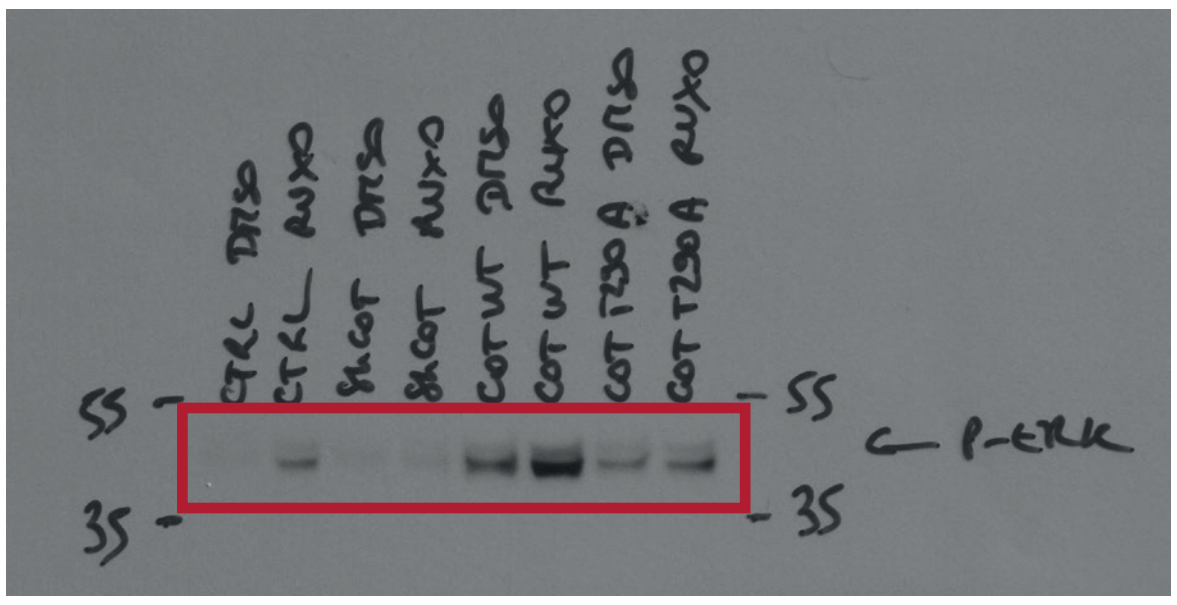

ERK

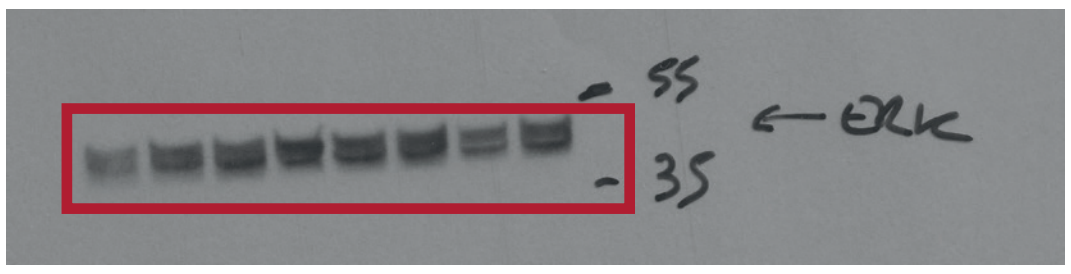

VINCULIN

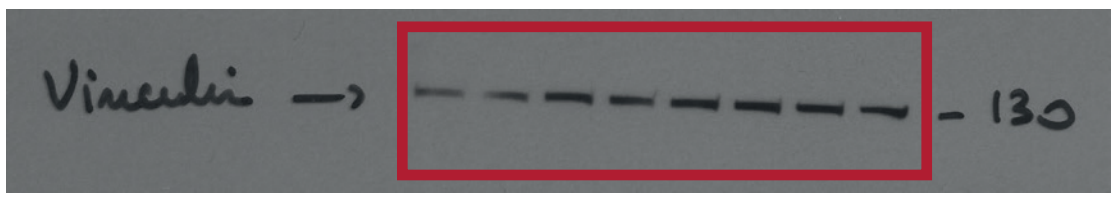

COT

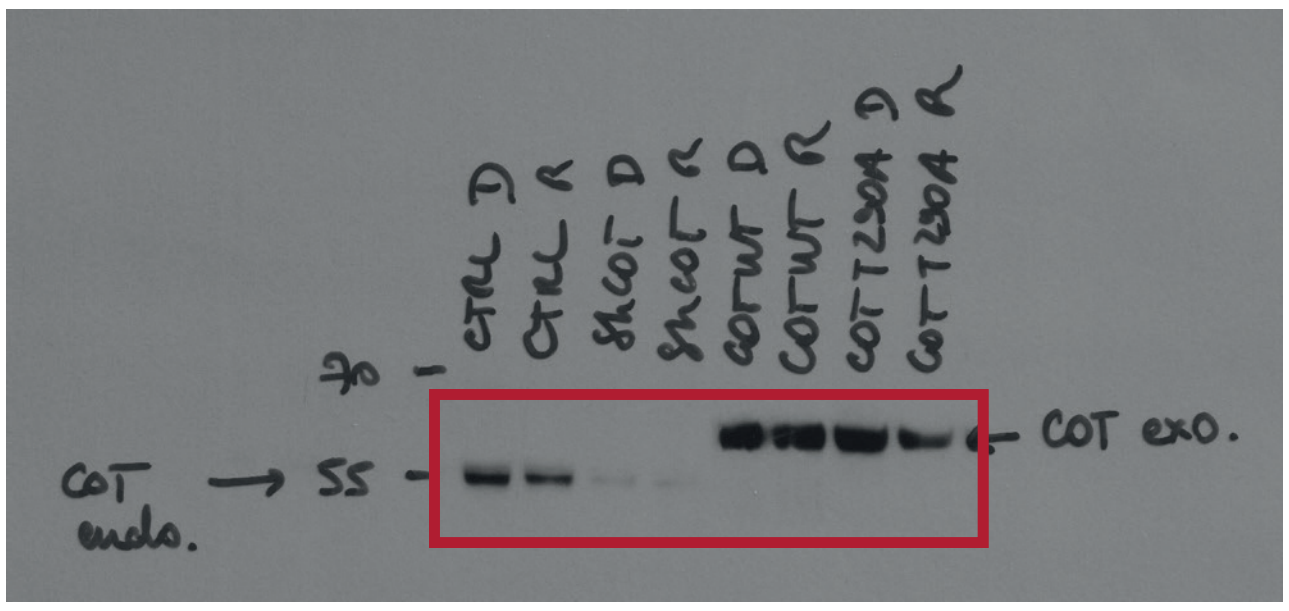

VINCULIN

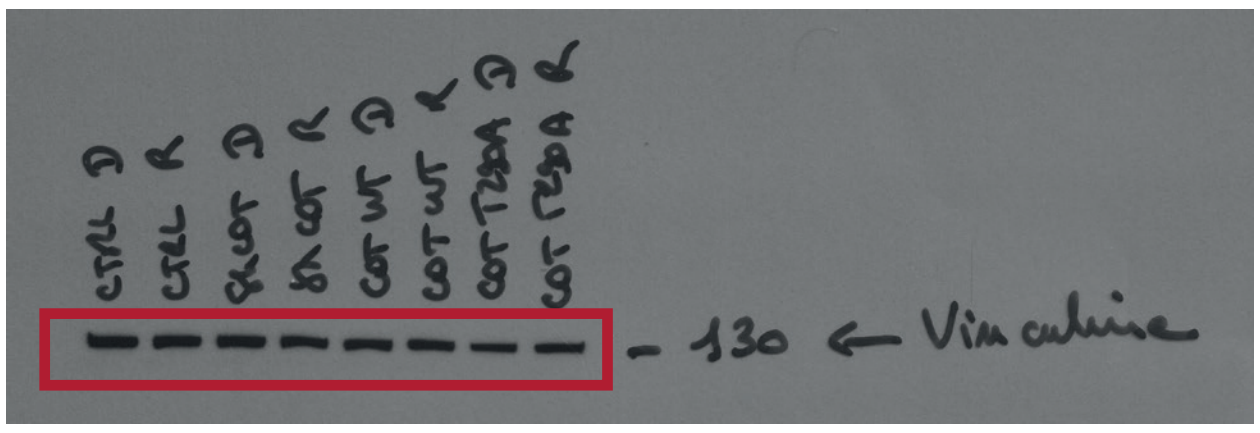

Figure 8B

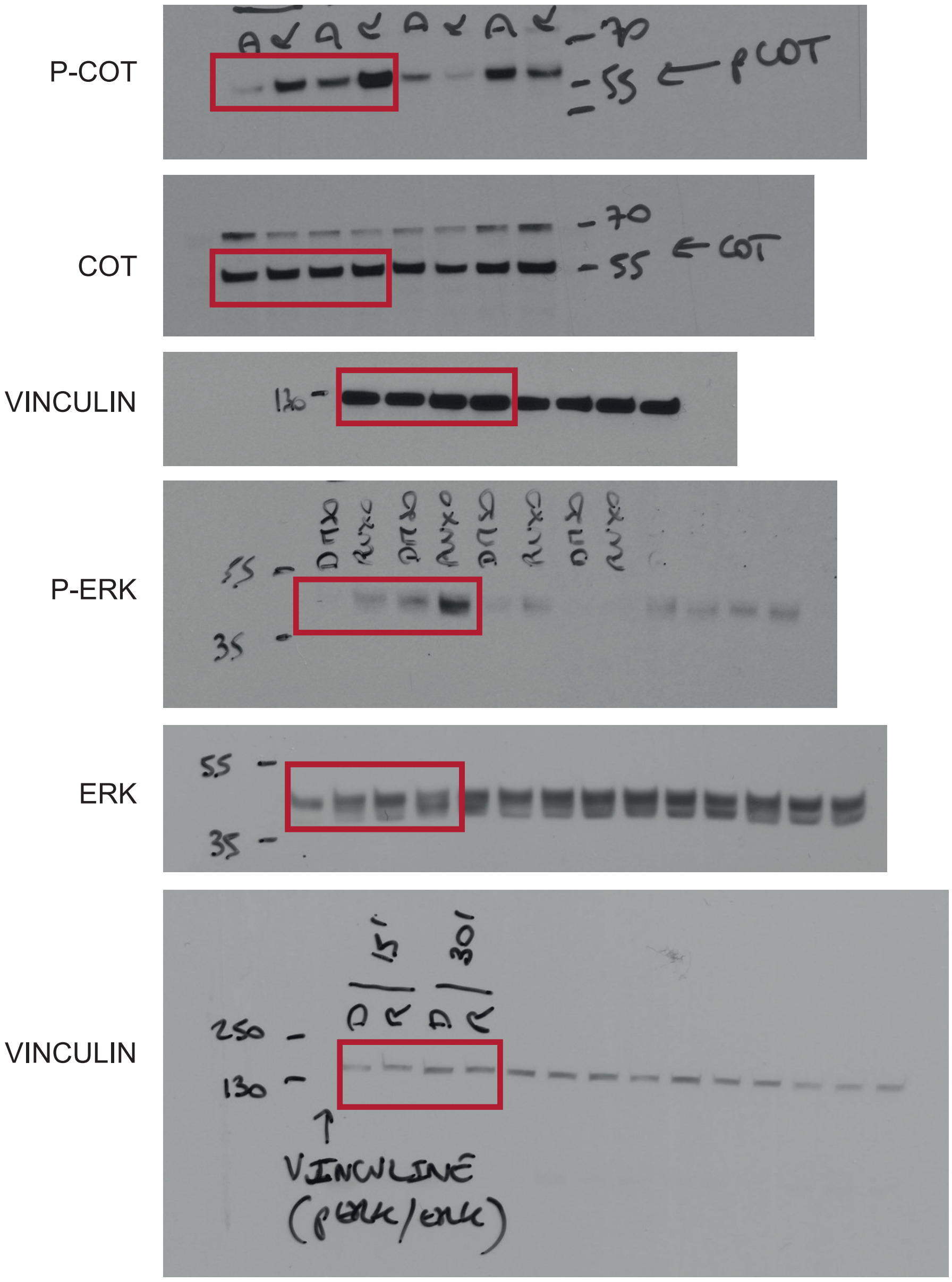

Figure 8C

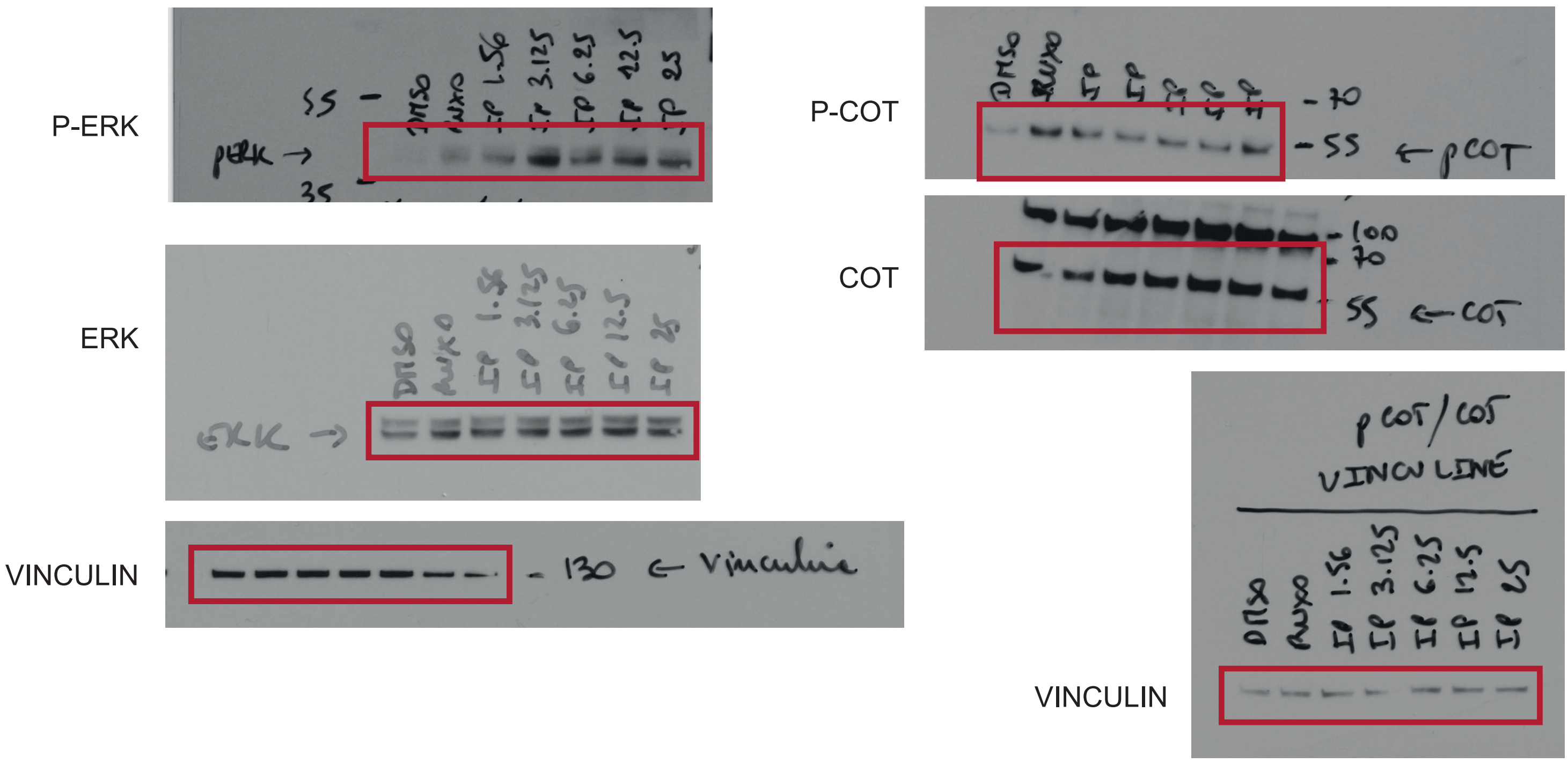

Figure S3B

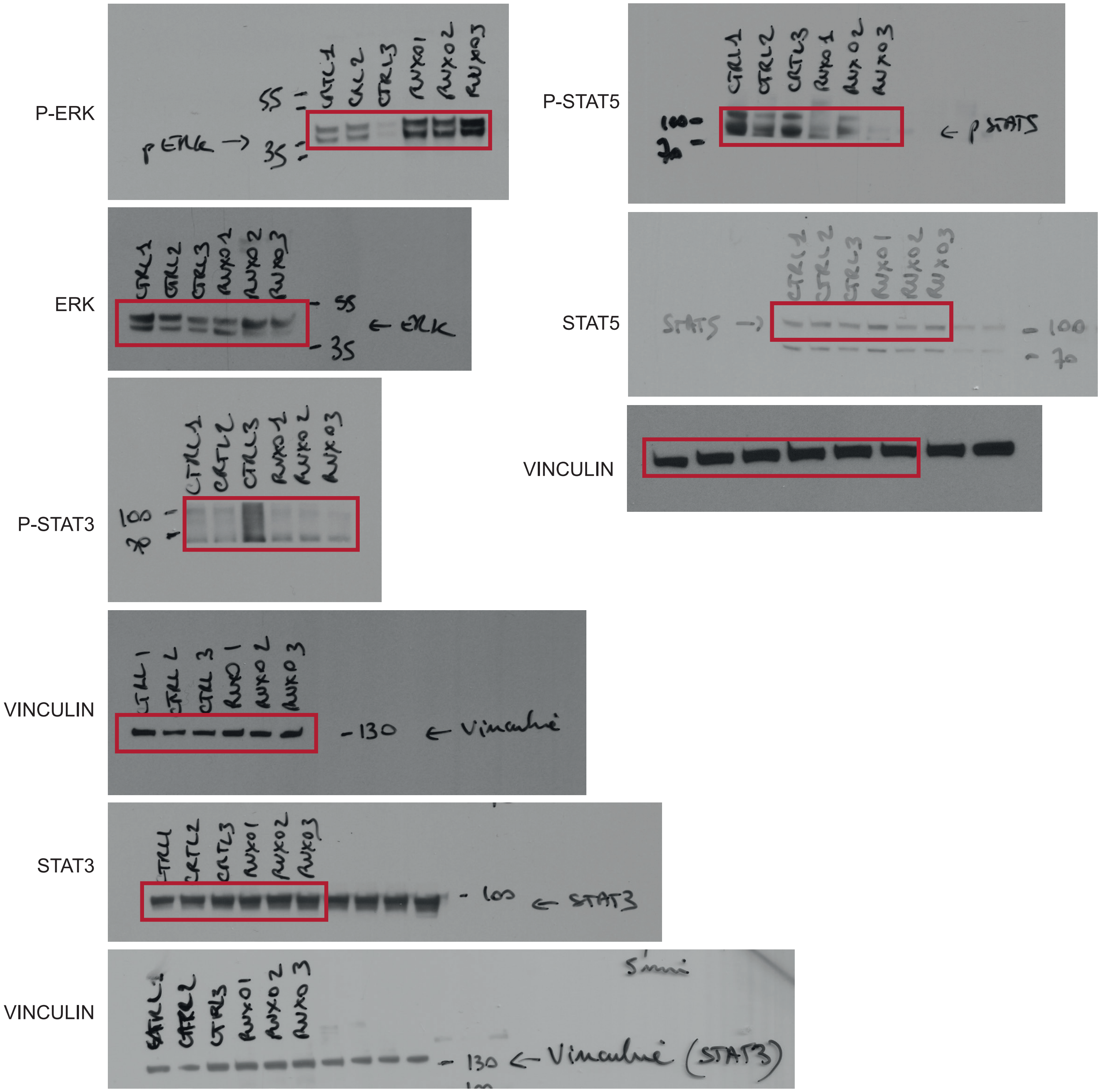
P-ERK
ERK
P-STAT3
VINCULIN
STAT3
VINCULIN
P-STAT5
STAT5
VINCULIN
